# Synthesis and Biological Evaluation of a Novel ^18^F-Labeled Radiotracer for PET Imaging of the Adenosine A_2A_ Receptor

**DOI:** 10.3390/ijms22031182

**Published:** 2021-01-25

**Authors:** Thu Hang Lai, Magali Toussaint, Rodrigo Teodoro, Sladjana Dukić-Stefanović, Mathias Kranz, Winnie Deuther-Conrad, Rareş-Petru Moldovan, Peter Brust

**Affiliations:** 1 Helmholtz-Zentrum Dresden-Rossendorf (HZDR), Department of Neuroradiopharmaceuticals, Institute of Radiopharmaceutical Cancer Research, Research Site Leipzig, 04318 Leipzig, Germany; r.teodoro@hzdr.de (R.T.); s.dukic-stefanovic@hzdr.de (S.D.-S.); mathias.kranz@uit.no (M.K.); w.deuther-conrad@hzdr.de (W.D.-C.); r.moldovan@hzdr.de (R.-P.M.); p.brust@hzdr.de (P.B.); 2 Department of Research and Development, ROTOP Pharmaka Ltd., 01328 Dresden, Germany; 3 PET Imaging Center, University Hospital of North Norway (UNN), 9009 Tromsø, Norway; 4 Nuclear Medicine and Radiation Biology Research Group, The Arctic University of Norway, 9009 Tromsø, Norway

**Keywords:** adenosine A_2A_ receptor, fluorine-18, positron emission tomography, tozadenant

## Abstract

The adenosine A_2A_ receptor (A_2A_R) has emerged as a potential non-dopaminergic target for the treatment of Parkinson’s disease and, thus, the non-invasive imaging with positron emission tomography (PET) is of utmost importance to monitor the receptor expression and occupancy during an A_2A_R-tailored therapy. Aiming at the development of a PET radiotracer, we herein report the design of a series of novel fluorinated analogs (**TOZ1**-**TOZ7**) based on the structure of the A_2A_R antagonist **tozadenant**, and the preclinical evaluation of [^18^F]**TOZ1**. Autoradiography proved A_2A_R-specific in vitro binding of [^18^F]**TOZ1** to striatum of mouse and pig brain. Investigations of the metabolic stability in mice revealed parent fractions of more than 76% and 92% of total activity in plasma and brain samples, respectively. Dynamic PET/magnetic resonance imaging (MRI) studies in mice revealed a brain uptake but no A_2A_R-specific in vivo binding.

## 1. Introduction

Adenosine is an endogenous ubiquitous molecule involved in many aspects of cellular physiology pertaining to neuronal activity, vascular function, platelet aggregation and immune cell regulation. It exerts its signaling by binding to four subtypes of purinergic P1 receptors, namely A_1_R, A_2A_R, A_2B_R and A_3_R, which are coupled to different G protein-mediated intracellular pathways. Among these receptors, the A_2A_R has emerged as an important non-dopaminergic target for the treatment of Parkinson’s disease (PD) based on its unique central nervous system distribution. The A_2A_R is highly enriched in the dorsal and ventral striatum, where it is involved in the indirect basal ganglia pathway and can form heteromeric complexes with other G protein-coupled receptors, including dopamine D_2_, glutamate mGluR_5_, cannabinoid CB_1_ and A_1_ [1,2,3,4]. Based on the co-localization and antagonistic interaction between A_2A_R and D_2_R, the beneficial effects of the A_2A_R blockade have been demonstrated in several clinical trials for treatment of PD. The adjunctive treatment with A_2A_R antagonists to the established levodopa (L-DOPA) therapy reduces postsynaptic effects of dopamine depletion, subsequently diminishing motor deficit of PD [5]. Additionally, co-administration with a low dose of L-DOPA induces an improvement of motor symptoms and a reduction in adverse effects from long-term L-DOPA treatment including dyskinesia, on-time shortening and psychotic syndromes [6,7].

**Figure 1:**
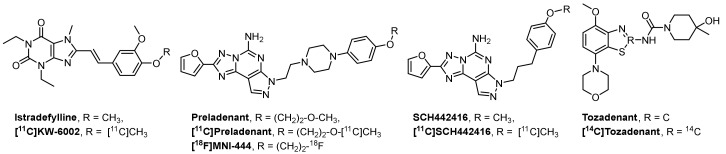
Representative adenosine A_2A_ receptor (A_2A_R) antagonists and the corresponding radiotracers in clinical trials.

Given the potential of an A_2A_R tailored-therapy to overcome the standard-treatment resistance several drugs have been investigated in clinical trials, such as **istradefylline**, **vipadenant**, **preladenant** and **tozadenant** (Figure 1). The xanthine derivate **istradefylline** (KW-6002, Nourianz^®^) from Kyowa Hakko Kirin Co. Ltd. (Tokyo; Japan) was the first A_2A_R antagonist approved by the U.S. Food and Drug Administration (FDA) as adjunctive anti-parkinsonian medication to L-DOPA/carbidopa treatment in patients experiencing “off” episodes [8,9,10]. Another selective and potent A_2A_R antagonist is the benzothiazole derivative **tozadenant** (SYN-115) [11]. Several clinical trials have been performed to assess the efficacy and safety dose ranges of **tozadenant** in PD patients. Although a positive off-time shortening from 2.5 h to 1 h was observed after a twice daily dose of 120 mg of **tozadenant**, a placebo-controlled phase III study (NCT02453386), designed to evaluate **tozadenant** in PD patients taking L-DOPA and experiencing motor fluctuations, was discontinued due to serious adverse effects. 

Positron emission tomography (PET) imaging of A_2A_R is regarded as a useful tool to advance our understanding of the functional role of the A_2A_R in healthy and diseased brains [12]. A selective A_2A_R PET radiotracer could be used to assess dose-dependent occupancy of the receptor population by drugs and to correlate with the efficacy of the potential tailored-therapy. Several ^11^C-labeled A_2A_R PET radiotracers have been developed and evaluated in clinical trials, such as [^11^C]**KW-6002**, [^11^C]**preladenant** and [^11^C]**SCH442416** (Figure 1) [13]. To date, [^18^F]**MNI-444** (Figure 1) is the only ^18^F-labeled radiotracer evaluated in healthy subjects with an excellent ratio of specific-to-nonspecific binding, reflecting a high dynamic range for e.g., receptor occupancy studies [14]. However, this radiotracer presents drawbacks limiting its routine clinical use due to relative slow kinetics, which may require too-long scan times.

To assess the suitability of PET for detection of changes in the availability of A_2A_R in PD, our group performed dynamic PET studies in a rotenone-based mouse model of Parkinson’s disease with [^18^F]**MRS5425**, (also known as [^18^F]**FESCH**) [15,16]. However, the study yielded inconclusive data, at least in part due to the presence of a non-negligible fraction of a brain-penetrating radiometabolite [17]. Therefore, we selected **tozadenant** as lead compound for the development of an A_2A_R-specific radiotracer based on its high affinity and its extensive evaluation in clinical trials. Although the development of **tozadenant** as a therapeutic drug has been discontinued due to adverse effects in a phase III clinical trial, this does not exclude the potential suitability of this A_2A_R ligand as diagnostic radiopharmaceutical. Due to the high sensitivity of PET scanners, the in vivo concentration of diagnostic tracers (between 1.10^−9^ and 1.10^−12^ M) is many orders of magnitude lower than the concentration applied in pharmacological studies. Based on the half-life of fluorine-18 (t_1/2_ = 109.7 min), very attractive for distribution to various PET centers after production, and its low positron energy (635 keV) facilitating high imaging resolution, we initiated our research with the goal to develop an ^18^F-labeled radiotracer with desirable physiochemical and pharmacokinetic properties. 

**Figure 2:**
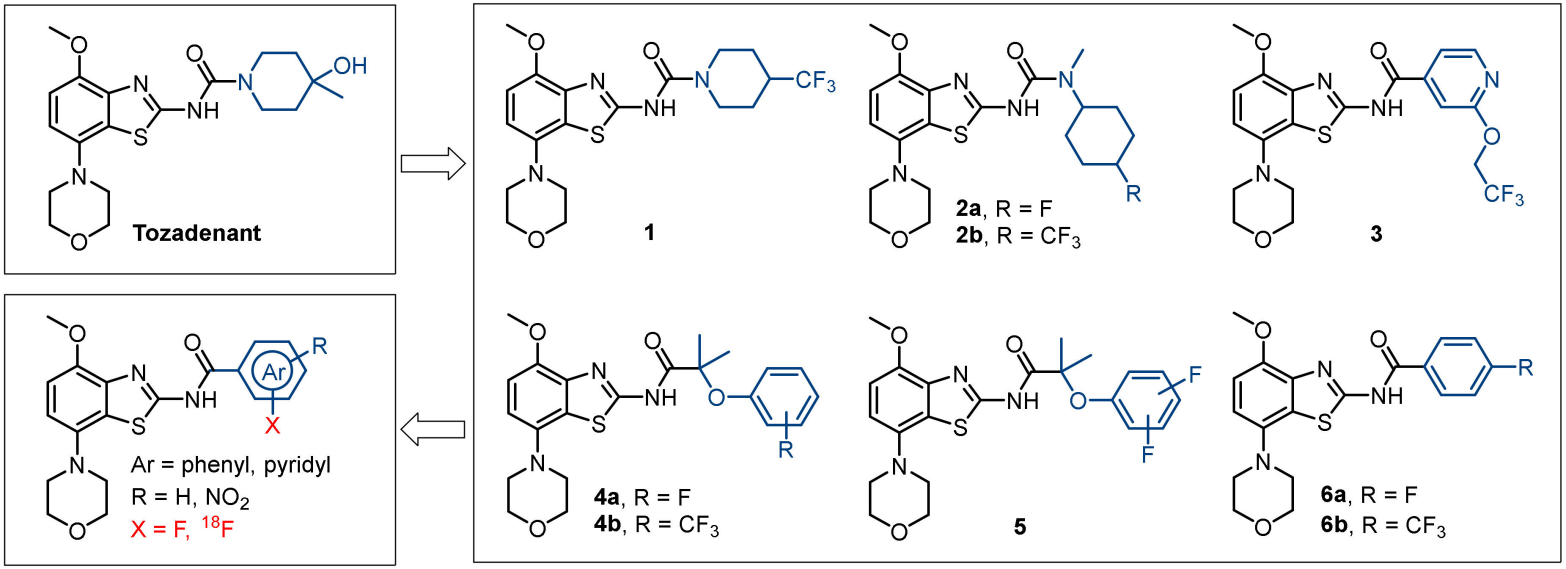
Design of a novel ^18^F-labeled radiotracer based on the lead compound **tozadenant** and its fluorinated derivatives.

Several morpholinobenzo[*d*]thiazol derivatives of **tozadenant** containing fluorine in aliphatic (**1**-**3**) and aromatic (**4**–**6**) positions were recently developed (Figure 2) [18,19,20,21,22]. However, we did not focus on A_2A_R ligands containing trifluoromethyl groups due to major limitations of the available labeling methods implicating low radiochemical yields and molar activities [23]. Therefore, the primary idea of our concept relies on the hypothesis that the introduction of a fluoroaryl moiety can potentially increase the radiochemical yields and the molar activities based on known radiolabeling strategies [24,25]. Furthermore, it could enhance the binding affinity via formation of π-interactions, hydrogen bonds or hydrophobic interactions with amino acid residues in the binding site of the A_2A_R. In particular for PET radiotracer development, we also investigated fluoropyridyl groups in the **tozadenant** scaffold since pyridine substituted with fluorine in positions 2 and 4 favor the radiofluorination via the aromatic nucleophilic substitution of known leaving groups [24,25]. Another consideration is based on the impact of different isomers on the binding affinity as shown for compound **4a** (*K*_i_(*h*A_2A_R) = 3.4 nM, 14 nM and 21 nM for *para*-F, *meta*-F and *ortho*-F, respectively) [22]. 

Herein, we describe the modification of **tozadenant** at the piperidine subunit with well tolerated fluoroaryl moieties based on compound **6a** (*K*_i_(*h*A_2A_R) = 2.0 nM) [18]. The binding affinities towards the A_2A_R and the selectivity towards the A_1_R subtype of the novel derivatives were determined and further used as pass–fail criteria (binding affinity toward *h*A_2A_R ≤ 2 nM, selectivity *h*A_2A_R/*h*A_1_R ≥ 200) for ^18^F-labeled radiotracer development. Finally, biological investigations of the selected candidate were performed to assess in vivo its potential for PET imaging of A_2A_R in the brain.

## 2. Results and Discussion

### 2.1. Chemistry

**Scheme 1:**
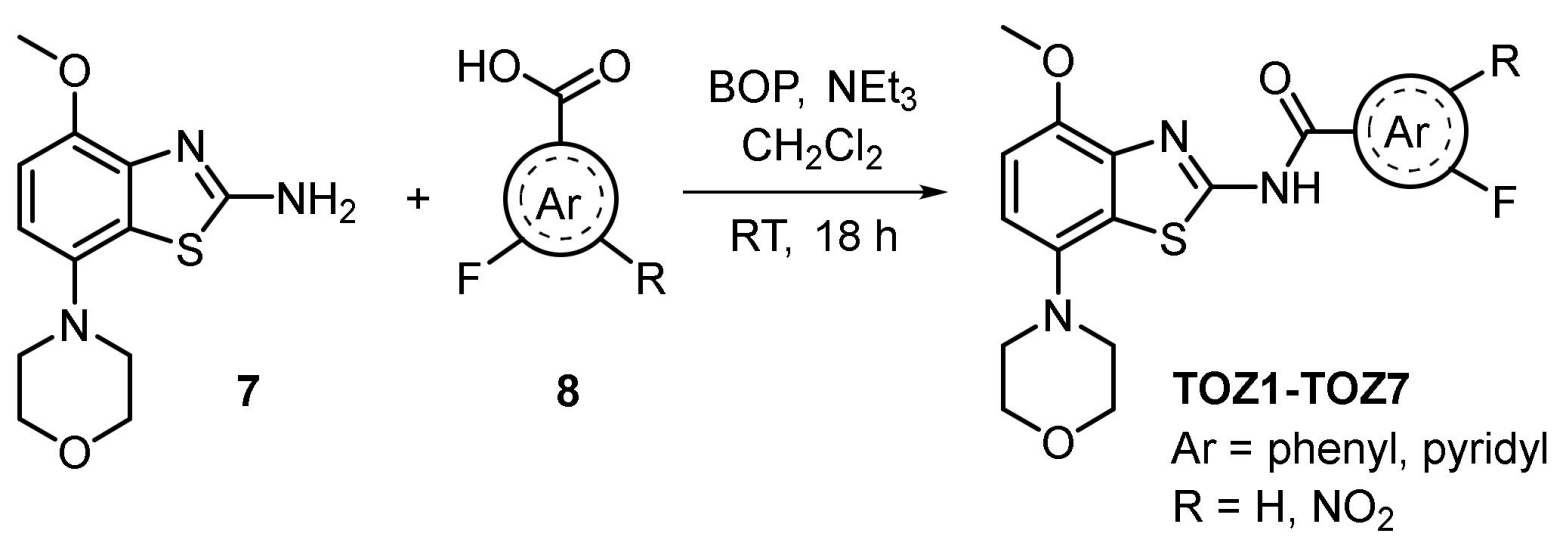
Synthesis of novel fluorinated **TOZ** derivatives.

The synthesis of the novel fluorinated derivatives was performed by an amide coupling reaction (Scheme 1). The commercially available 4-methoxy-7-morpholinobenzo[d]thiazol-2-amine **7** was reacted with the corresponding fluorobenzoic acid or fluoropyridine carboxylic acid **8** in the presence of (benzotriazol-1-yloxytris(dimethylamino)phosphonium hexafluorophosphate) (BOP) as coupling agent resulting in the desired fluorinated derivatives **TOZ1**-**TOZ7** with 29–86% yields. The nitro precursor **9** was synthesized by an amide coupling reaction with 1-[bis(dimethylamino)methylene]-1*H*-1,2,3-triazolo[4,5-*b*]pyridinium 3-oxide hexafluorophosphate (HATU) instead of BOP to obtain slightly higher yields (37% vs. 25%).

### 2.2. Structure–Activity Relationship Studies

**Figure 3:**
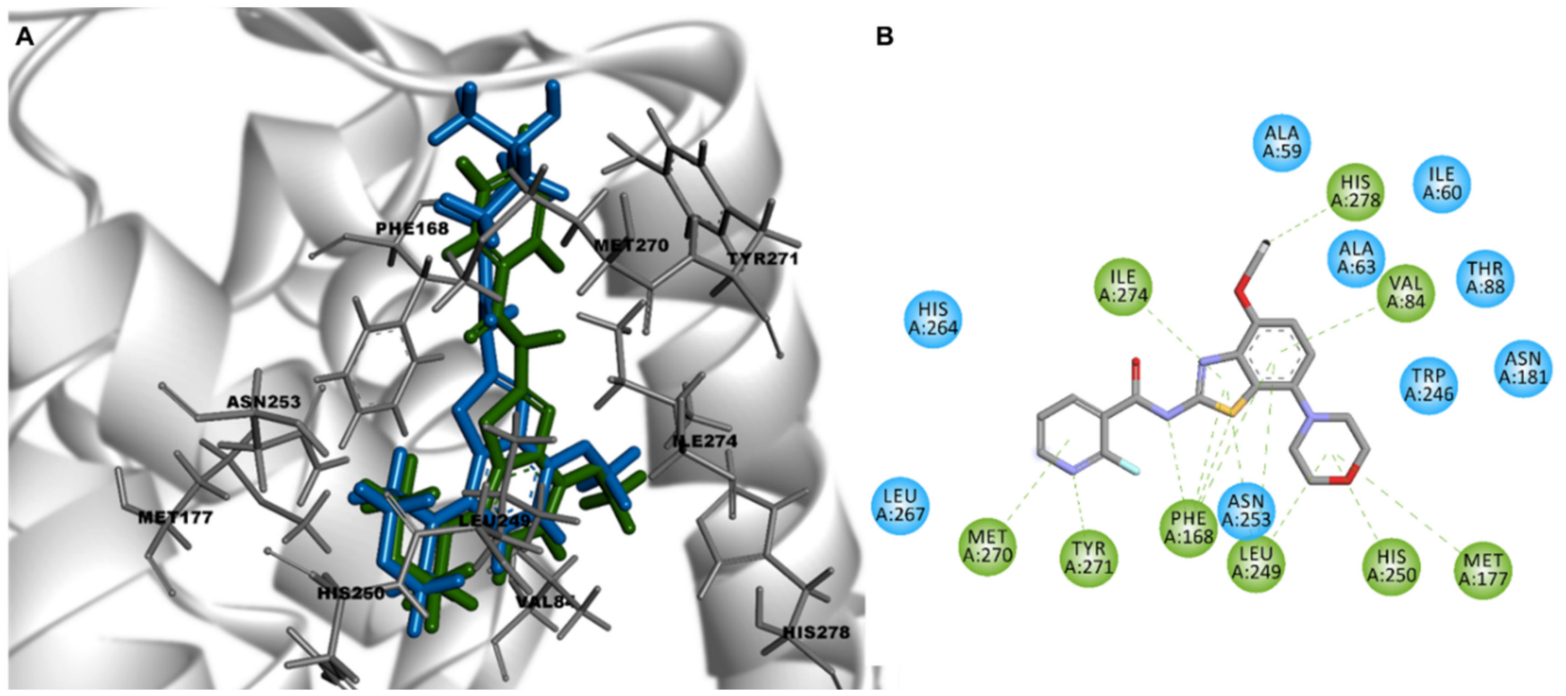
(**A**) Overlay of the docking poses of **tozadenant** (blue) and **TOZ1** (green) in the binding site of crystal structure of A_2A_R (Protein Data Bank (PDB) ID: 4EIY) and (**B**) 2D interaction diagram of **TOZ1** with key interactions in green.

Preliminary quantitative structure–activity relationship (QSAR) modelling studies were carried out to predict binding poses and intermolecular interactions responsible for the receptor binding affinity (*K*_i_) determined by radioligand binding assays (Figure 3). The X-ray crystallographic chimeric protein structure of A_2A_R–BRIL in complex with the antagonist **ZM241385** (Protein Data Bank (PDB) ID: 4EIY) was selected for QSAR modelling due to the highest resolution (1.8 Å) of all available non-thermostabilized structures [26]. The benzothiazole core of **tozadenant** is located in the orthosteric binding site and is stabilized by hydrophobic aromatic π–π stacking interaction with Phe168 and van der Waals interactions with Leu249 and Ile274 [22]. The methoxy and carbonyl group form hydrogen bonds with Asn253 and Tyr271, respectively. The morpholine group is surrounded by several hydrophobic residues, such as Ala63, Ile66, Ala81 and Val84. Individual docking poses of all fluorinated ligands based on the morpholinobenzo[*d*]thiazol-2-amine scaffold show identical key binding interactions with Phe168, Leu249 and Ile274. The replacement of the 4-hydroxy-methylpiperidine group of **tozadenant** by fluorinated (hetero)arenes has no relevant influence on additional intermolecular interactions. Furthermore, the modified part of the compound is exposed to the solvent front (Figure 3). Therefore, it is assumed that further chemical modifications of the (hetero)arene group may not result in more affine A_2A_R ligands.

The structures of the final products prepared in this study along with in-house determined in vitro binding affinities towards the A_2A_R and A_1_R subtypes are given in Table 1. Fluorine was introduced by fluoropyridyl (**TOZ1**-**TOZ4**) and fluorobenzyl (**TOZ5**-**TOZ7**) groups into the morpholinobenzo[*d*]thiazol-2-amine scaffold of **tozadenant**. Although all these synthesized derivatives exhibit a high A_2A_R binding affinity, a decrease in the selectivity towards the A_1_R was observed for mono-substituted fluoropyridyl derivatives (**TOZ1**-**TOZ4**) in comparison to fluorobenzyl derivatives (**TOZ5**, **TOZ6**). Furthermore, a remarkable 13-fold decreased A_1_R binding affinity was obtained by addition of a second substituent into the benzyl ring (**TOZ5** vs. **TOZ7**). The position of the fluorine seems also to have an influence on the binding affinity towards the A_2A_R and the A_1_R. Thus, the fluorination in para position to the carboxamide moiety (**TOZ1**, **TOZ5**) led to a five-fold increased binding affinity compared to the fluorine in ortho-position (**TOZ2**, **TOZ6**). Among our fluorinated derivatives, the most affine A_2A_R ligand **TOZ1** was selected for radiofluorination to investigate its in vitro binding pattern and its in vivo brain pharmacokinetic.

### 2.3. Radiochemistry

**Figure 4:**
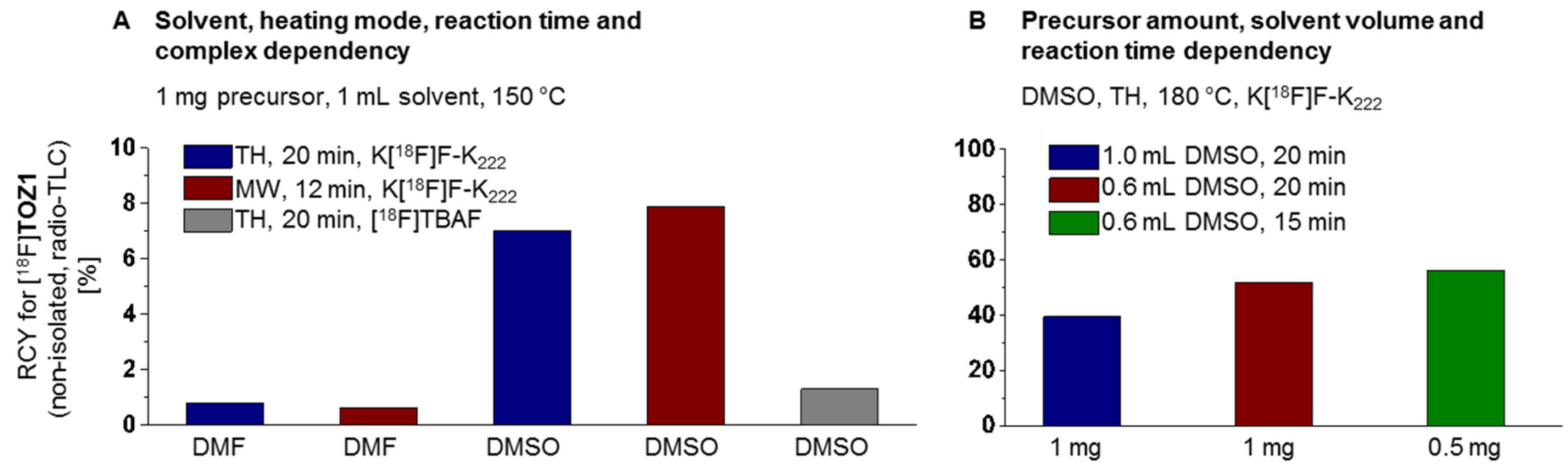
Investigated reaction conditions for the radiosynthesis of [^18^F]**TOZ1**: (**A**) solvent, heating mode (RCY = radiochemical yield, TH = thermal heating, MW = microwave heating), reaction time, complex and (**B**) precursor amount, solvent volume, reaction time.

For the establishment of the radiosynthesis of [^18^F]**TOZ1**, the reaction conditions were systematically optimized by varying the base, the solvent, the amount of the nitro precursor **9**, the reaction time, the temperature and the mode of heating (conventional vs. microwave, *n* = 1). Aliquots of the reaction mixtures were analyzed by radio-thin-layer chromatography (TLC) (Figure 4). First, the impact of the solvent (*N,N*-dimethylformamide (DMF) vs. dimethyl sulfoxide (DMSO)) and the heating mode were tested with 1 mg of the precursor **9** at 150 °C. Radiochemical yields of lower than 1% of [^18^F]**TOZ1** were observed for the conventional as well as microwave-assisted radiofluorination using DMF as solvent. Although the microwave-assisted radiofluorination (150 °C, 100 W, 12 min) with DMSO afforded [^18^F]**TOZ1** slightly higher radiochemical yields (~8%), the conventional thermal heating approach was further investigated because currently no microwave device-coupled automated synthesis module is commercially available. Moreover, by using [^18^F]TBAF instead of a K[^18^F]F–K_222_ complex in DMSO (conventional heating, 150 °C, 20 min) resulted in a decrease in the radiochemical yield to 1% of [^18^F]**TOZ1**.

Thereafter, we attempted to further investigate the impact of the temperature on our procedure. The radiofluorination of the nitro precursor **9** (1 mg) was then performed at 180 °C in DMSO (1 mL). The use of the higher temperature led to a significant increase in the radiochemical yield to 40% of [^18^F]**TOZ1**. The radiochemical yield could be further optimized to 52% by reducing the DMSO reaction volume from 1 mL to 0.6 mL. Furthermore, the amount of the nitro precursor **9** was reduced from 1 mg to 0.5 mg without considerable impact on the radiochemical yield (52% vs. 56%). Herein, the use of low precursor amounts is beneficial for the isolation of [^18^F]**TOZ1** due to the similarity of the chromatographic behavior as previously shown for other radiotracers [27,28,29].

**Figure 5:**
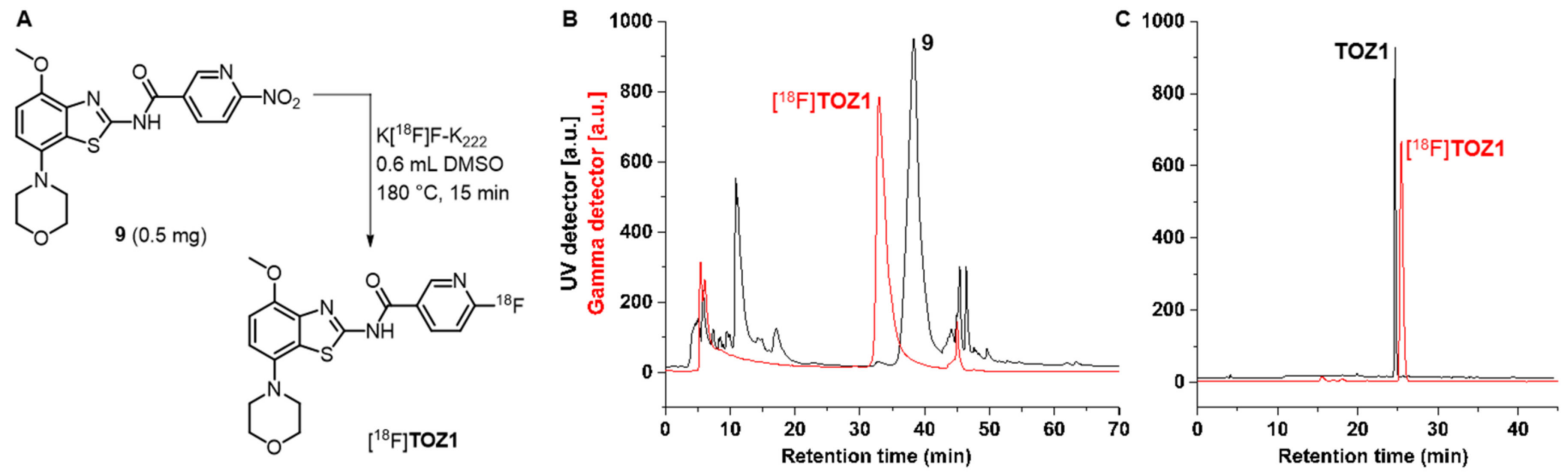
(**A**) Radiosynthesis of [^18^F]**TOZ1** by radiofluorination of the nitro precursor **9**. (**B**) Representative radio- and UV-chromatograms obtained for the isolation of [^18^F]**TOZ1** by semi-preparative reversed-phase (RP)-HPLC (ReproSil-Pur 120 C18-AQ (250 × 10 mm), 36% acetonitrile (MeCN)/H_2_O/0.05% trifluoroacetic acid (TFA), flow rate: 4 mL/min); and (**C**) radio- and UV-chromatograms of formulated [^18^F]**TOZ1** co-injected with the corresponding reference compound **TOZ1** (ReproSil-Pur 120 C18-AQ column (250 × 4.6 mm), 10-90-10% MeCN/20 mM NH_4_OAc_aq._ flow rate: 1 mL/min).

The optimized procedure for the radiosynthesis of [^18^F]**TOZ1** is summarized in Figure 5A. In brief, the anhydrous K[^18^F]F–K_222_ complex was stirred with the nitro precursor **9** (0.5 mg) in 0.6 mL DMSO at 180 °C for 15 min. The non-isolated radiotracer [^18^F]**TOZ1** was observed in a radiochemical yield of 61 ± 10% (radio-TLC, n = 8) and 66 ± 17% (radio-high-performance liquid chromatography (radio-HPLC), n = 5). Afterwards, [^18^F]**TOZ1** was isolated via semi-preparative reversed-phase HPLC (RP-HPLC) with a retention time of about 31 min (Figure 5B), which was required to separate the radiotracer from the nitro precursor **9**. The final purification and concentration proceeded smoothly by loading on a preconditioned reversed phase-solid phase extraction (RP-SPE) cartridge and subsequent elution of [^18^F]**TOZ1** with absolute ethanol (EtOH). For biological investigation, the ethanolic solution containing [^18^F]**TOZ1** was reduced under a gentle argon stream at 70 °C. [^18^F]**TOZ1** was finally formulated in isotonic saline containing < 10% of EtOH (*v*/*v*). Starting with activities ranging from 2–3 GBq, [^18^F]**TOZ1** was successfully obtained with a radiochemical yield of 37 ± 7% (*n* = 3, end of bombardment = EOB), a high radiochemical purity (≥99%) and molar activities in the range of 62–72 GBq/µmol (end of synthesis = EOS) in a total synthesis time of about 114 ± 3 min.

The manual radiosynthesis was transferred to the automated synthesis module Synchrom R&D EVO-III Raytest (see Supplementary Materials, Figure S1-1). Starting with activities ranging from 3–5 GBq, [^18^F]**TOZ1** was successfully obtained with a radiochemical yield of 33 ± 10% (*n* = 2, EOB), a high radiochemical purity (≥99%) and molar activities in the range of 40–44 GBq/µmol (EOS) in a total synthesis time of about 115 ± 5 min. Radio-HPLC analysis of the final product co-eluted with the corresponding reference compound **TOZ1** confirmed the identity of the radiotracer (Figure 5C).

The stability of [^18^F]**TOZ1** was proven in all tested media (saline, phosphate-buffered saline (PBS) and *n*-octanol) at 37 °C up to 60 min. The distribution coefficient (Log*D*_7.4_) in the *n*-octanol–PBS system was experimentally determined by the shake-flask method. Observing a Log*D*_7.4_ value of 1.74 ± 0.08 (*n* = 3) for [^18^F]**TOZ1**, we assume that a significant passive diffusion through the blood-brain barrier (BBB) can be expected for the radiotracer [30,31,32].

### 2.4. Metabolite Analysis

**Figure 6:**
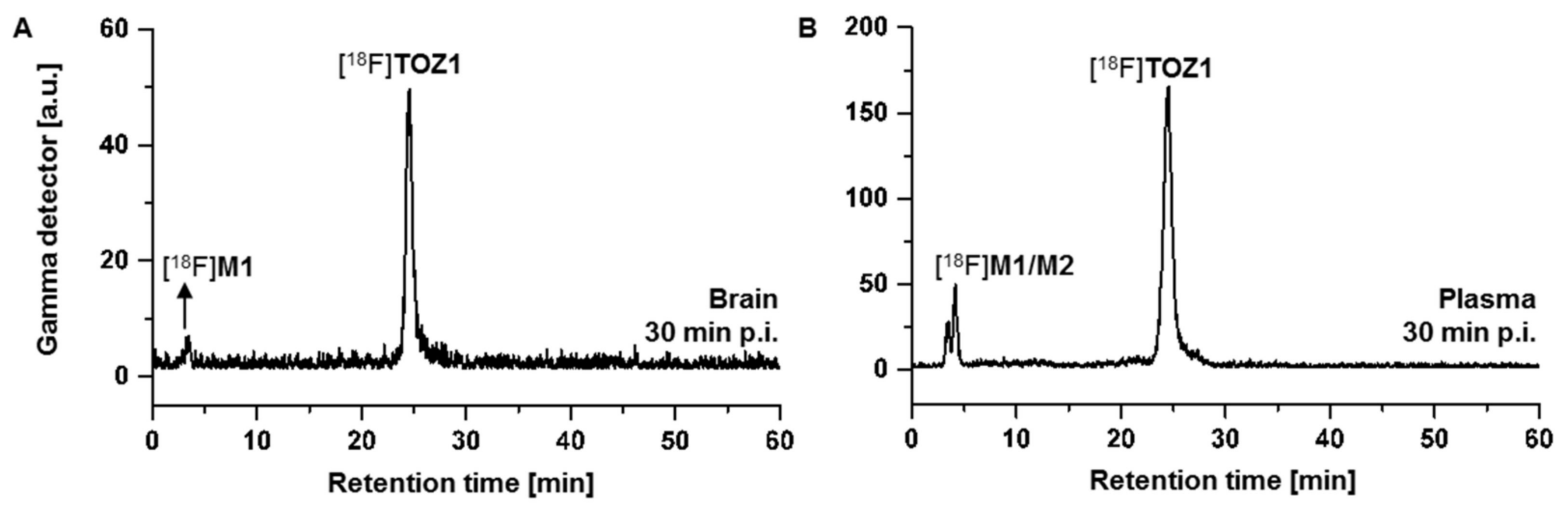
Representative radio-chromatograms of the in vivo metabolism study of extracted mouse (**A**) brain and (**B**) plasma samples at 30 min post injection (p.i.) of [^18^F]**TOZ1** (ReproSil-Pur 120 C18- AQ column (250 × 4.6 mm, 5 µm), 10-90-10% MeCN/20 mM NH_4_OAc_aq_., flow rate: 1 mL/min).

Radiometabolite analysis of [^18^F]**TOZ1** was performed in plasma samples and brain homogenates obtained at 30 min after radiotracer injection in CD-1 mice (*n* = 2). Recovery efficiencies of extracted activity were 87–90% for brain and plasma samples, respectively. [^18^F]**TOZ1** demonstrated a high in vivo stability in plasma with a parent fraction of 76% in mice (Figure 6B). Two hydrophilic radiometabolites were detected in plasma samples, that most likely result from defluorination or deamidation as previously reported in an in vivo study of [^14^C]**tozadenant** [33]. The metabolism of halogenated analogs of nicotinic acid was intensively studied by several groups and similar results were observed for [^18^F]**TOZ1** [34,35]. Based on these studies, the metabolism could be explained by the metabolic degradation of the [^18^F]fluoronicotinic acid and the formation of [^18^F]fluoronicotinate mononucleotide, which is then converted to [^18^F]fluoronicotinate adenine dinucleotide and [^18^F]fluoronicotinamide adenine dinucleotide [36].

Although one of these radiometabolites is brain-penetrant ([^18^F]**M1**: t_R_ = 3 min, Figure 6A), the majority of activity accumulating in the brain after injection of [^18^F]**TOZ1** corresponds to the parent compound (92%). In comparison to the A_2A_R radiotracer [^18^F]**FESCH** (15 min post injection (p.i.): parent fractions of 71% and 41% in brain and plasma samples, respectively) [17], [^18^F]**TOZ1** seems to have a remarkable metabolic stability in mice.

### 2.5. Autoradiography Study

**Figure 7:**
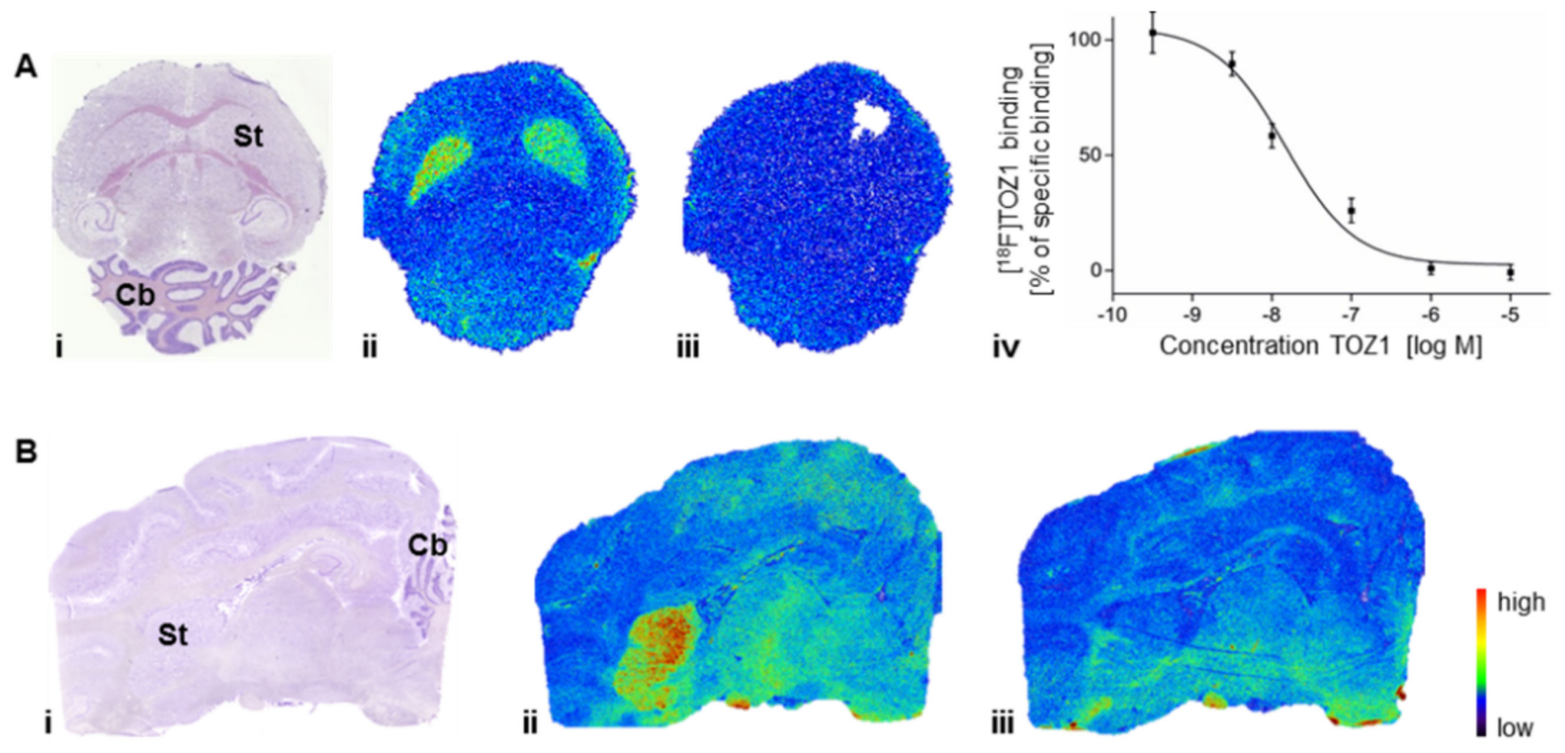
Representative autoradiographic images of (**A**) the transversal plane of mouse and (**B**) the sagittal plane of pig brain slices after incubation with 1.1 nM [^18^F]**TOZ1**: (i) Nissl staining; (ii) total binding; and (iii, iv) nonspecific binding in the presence of 1 µM **ZM241385**; St = striatum, Cb = cerebellum.

The in vitro studies were performed to determine the binding pattern of [^18^F]**TOZ1** in mouse and pig brain (Figure 7). The highest density of binding sites of [^18^F]**TOZ1** in mouse and pig brain was observed in the striatum, an A_2A_R-rich region. In A_2A_R-poor regions, such as cerebellum, midbrain, cortex or thalamus, only negligible binding was detected. Approx. 60% of the binding of 1 nM [^18^F]**TOZ1** in mouse and pig striatum could be displaced by co-incubation with 1 µM **ZM241385**, an A_2A_R-selective antagonist. The binding sites in mouse striatum were further characterized by homologous competition, which revealed an equilibrium dissociation constant (*K*_D_) value of 23.0 ± 5.5 nM (n = 3) and a maximum receptor density (*B*_max_) value of 287 ± 104 fmol/mg wet weight (n = 3). In practice, a theoretical binding potential (BP = *B*_max_/*K*_D_) value estimated from in vitro measurements of greater than 10 is generally required for proper in vivo imaging [37]. [^18^F]**TOZ1** possesses an in vitro estimated BP value of ~12 in mice and of ~350 (*K*_D_: 1.0 nM, *B*_max_: 260–44 fmol/mg protein) [38,39], in human promising for in vivo application. For comparison, the well-characterized radiotracer [^11^C]**SCH442416** displays a satisfying BP value of 85 in human [40], which suggests the suitability of [^18^F]**TOZ1** for human application regarding its estimated binding potential.

### 2.6. PET Studies

**Figure 8:**
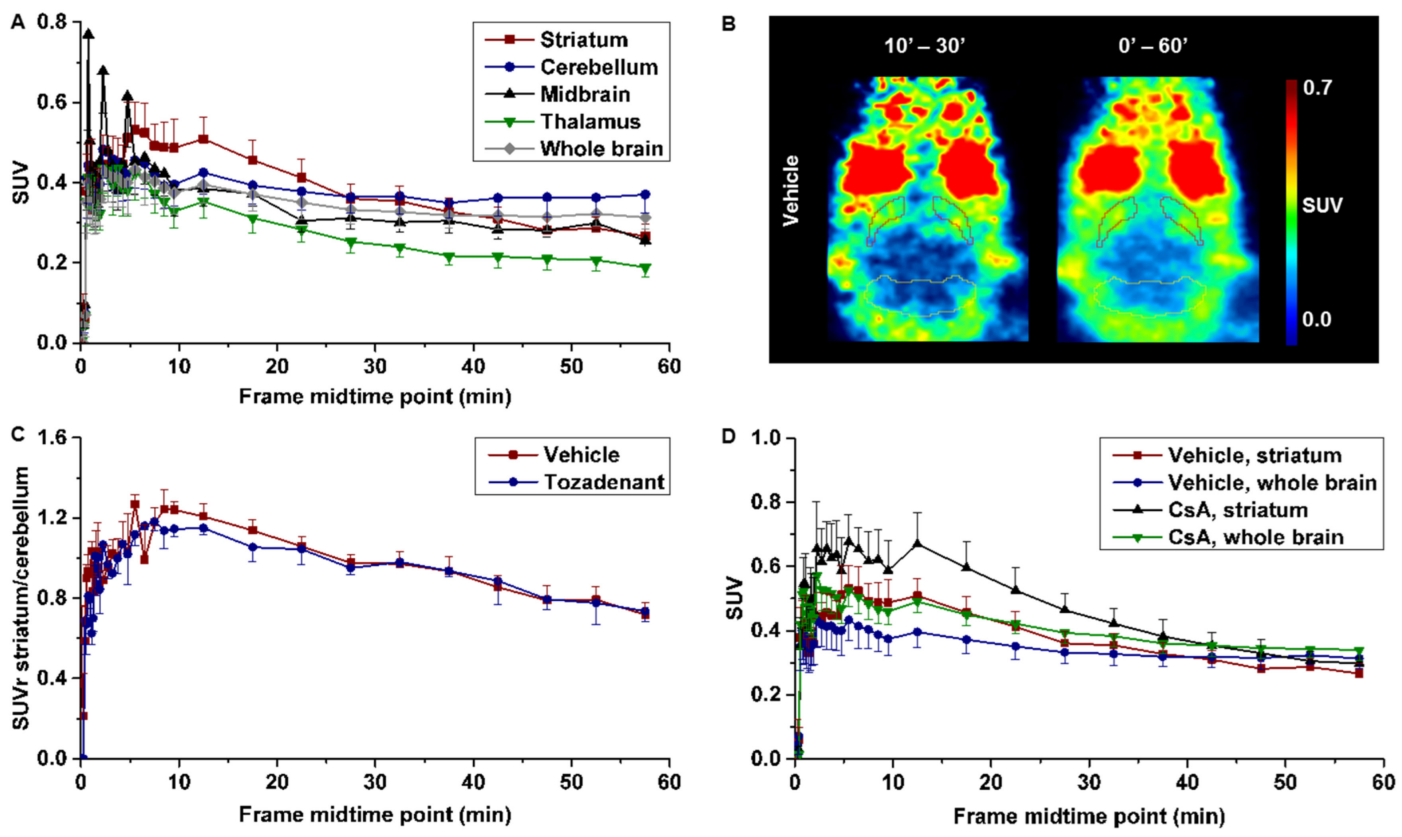
(**A**) Time–activity curves (TACs) of CD-1 mice in different brain regions after injection of [^18^F]**TOZ1** (*n* = 7); (**B**) representative horizontal positron emission tomography (PET) images after pre-treatment with vehicle; (**C**) TACs of standardized uptake value ratio (SUVr) of striatum-to-cerebellum after pre-treatment with vehicle (*n* = 7) or **tozadenant** (2.5 mg/kg, *n* = 3); and (**D**) TACs after pre-treatment with vehicle (*n* = 7) or **cyclosporine A** (CsA, 50 mg/kg, *n* = 4). Mean SUV ± SEM.

The pharmacokinetic of [^18^F]**TOZ1** was evaluated in healthy CD-1 mice under baseline and blocking condition. The initial brain uptake of [^18^F]**TOZ1** described by a standardized uptake value (SUV) of 0.4 between 1–5 min indicates a sufficient—although low—BBB permeability to the radiotracer followed by a slow washout depicted on Figure 8A. The regional time–activity curve (TAC) of the A_2A_R-rich region striatum presents a similar initial uptake to the A_2A_R-poor regions, such as cerebellum, midbrain, and thalamus (Figure 8A).

Nevertheless, a slightly higher accumulation in the striatum compared to cerebellum between 10 and 30 min p.i. indicates specific binding (Figure 8A,B). Although the signal-to-background ratio described by a maximum SUV ratio (SUVr) of striatum-to-cerebellum of 1.2 presumes a specific binding of [^18^F]**TOZ1** in vivo; this value is considerably lower than anticipated from the in vitro binding studies (total binding ratio striatum-to-cerebellum of 1.7). However, in vitro and in vivo binding parameters may differ due to the much higher complexity of the in vivo environment, such as the presence of endogenous ligands. The principal difference with regard to the binding process arises from the fact that binding assays in vitro are performed under equilibrium conditions, which strongly affects the rate constants *k*_on_ and *k*_off_ [41].

Accordingly, the target selectivity of [^18^F]**TOZ1** was investigated by pre-administration of the A_2A_R antagonist **tozadenant**, which could not reduce the accumulation of the activity in the brain in comparison to the vehicle (Figure 8C). By determination of the *K*_i_ value of **tozadenant** in mice, a 20–50-fold lower affinity than that known for human has been observed (*K*_i_ ~ 200 nM (*m*), 5 nM (*h*); unpublished data), which could explain the lack of blocking efficiency apart of the high nonspecific binding observed in the A_2A_R-poor region. Furthermore, it has to be noted that we have determined a high [^18^F]**TOZ1** selectivity towards the human A_1_R subtype (*h*A_2A_R/*h*A_1_R > 600).Thus, the discrepancies between the expected selectivity and our in vivo findings of [^18^F]**TOZ1** in mice could be related to the investigated animal model.

Further investigations were performed to elucidate if the low initial uptake could be related to the permeability-glycoprotein (P-gp) a drug efflux transporter restricting the entrance of several compounds such as chemotherapeutics (e.g., paclitaxel) [42]. After pre-administration of **cyclosporine A** (P-gp inhibitor) a tendency to a higher uptake was observed in the striatum and the whole brain, but it was not significant to ensure that [^18^F]**TOZ1** is a P-gp substrate (Figure 8D). Although the determined Log*D*_7.4_ of 1.74 ± 0.08 was in favor of BBB penetrance, this lipophilicity may also increase the probability of binding to hydrophobic proteins and thus decrease the fraction able to cross the BBB [43].

**Figure 9:**
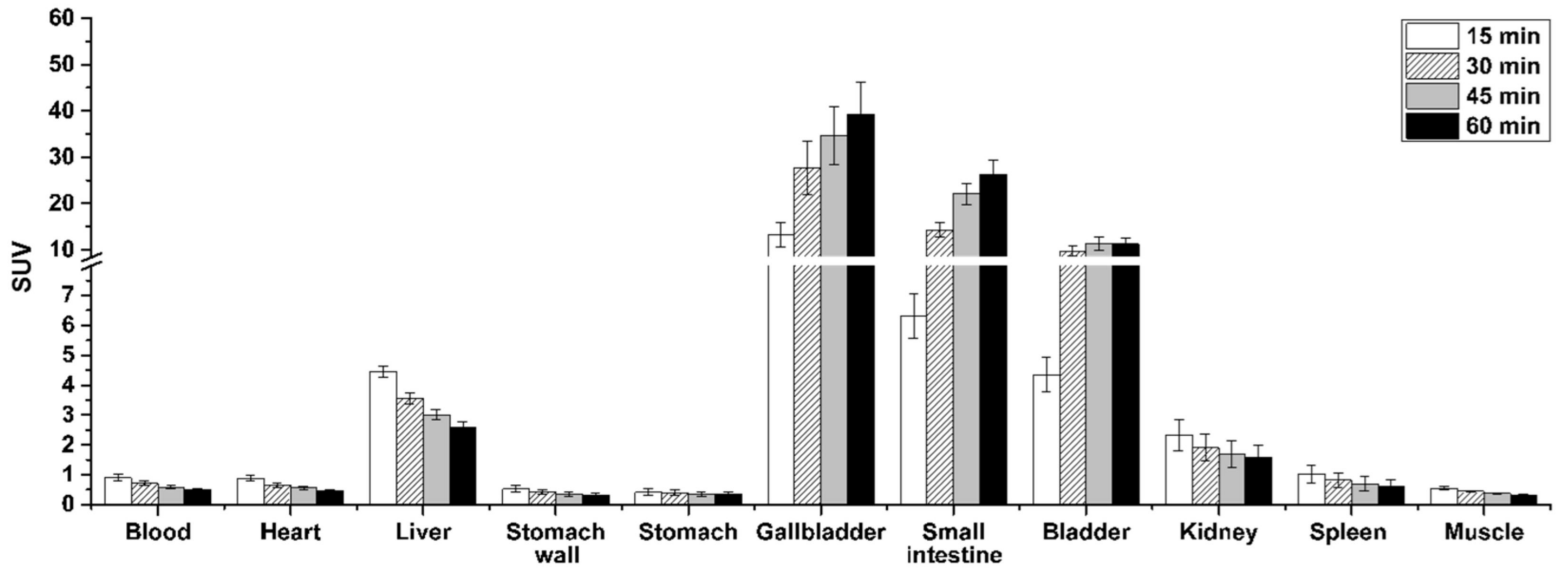
Biodistribution of [^18^F]**TOZ1** at different time points based on PET imaging (*n* = 7, mean SUV ± SEM).

Further analysis of the whole-body PET data revealed a low activity uptake in the heart, stomach, spleen and to a less extent in the liver (initial SUV < 5), while high accumulation occurred in the gallbladder (SUV_60 min_ 39.2), the small intestine (SUV_60 min_ 26.3) and the bladder (SUV_60 min_ 11.2) indicating a hepatobiliary and urinary excretion (Figure 9).

All these results raise the question of the suitability of the selected animal model for A_2A_R imaging. In previous studies, we already investigated another A_2A_R radioligand in mice: [^18^F]**FESCH**, an ^18^F-labeled derivative of the clinically used **SCH442416** [15,16,17]. [^18^F]**FESCH** presented an inferior metabolism (15 min p.i.: parent fractions of 71%) compared to [^18^F]**TOZ1**, but a good specific binding (maximum SUVr striatum-to-cerebellum of ~5), arguing for the suitability of the mouse model for A_2A_R radioligand characterization. However, the predictive power of such an animal model can differ from one class of compound to another. The homology sequences between the human and rat or mouse ADORA2A genes is about 84%, and can lead to variations in the receptor pharmacology with regards to the tested compound [44]. Stone et al. estimated in vitro a similar binding affinity of **NECA**, a nonselective A_2A_R agonist, in human, rat and mouse (*K_i_* = 9.0 nM (*h*), 8.3 nM (*r*), 9.0 nM (*m*)) but an almost two-times lower binding affinity of **theophylline**, a nonselective A_2A_R antagonist, in human compared to mouse and rat (*K_i_* = 7.8 nM (*h*), 15.9 nM (*r*), 12.9 nM (*m*)) accompanied by variations in the selectivity towards the A_1_R subtype which could also impact the in vivo imaging quality [45]. In conclusion, the in-house determined *K_i_* values by radioligand binding assay of [^18^F]**TOZ1** towards *h*A_2A_R and *m*A_2A_R (0.85 nM vs. 10 nM), tend to prove that the mouse model is suitable for the characterization of our **tozadenant** derivatives. However, further studies regarding the selective binding of [^18^F]**TOZ1** in mice are needed to validate its potential for brain imaging of the A_2A_R in humans.

## 3. Materials and Methods

### 3.1. General Information

All chemicals and reagents were purchased from commercially available sources and used without further purification. Moisture-sensitive reactions were conducted under dry argon with oven-dried glassware and anhydrous solvents. Reaction progress was monitored by thin-layer chromatography (TLC) using Alugram^®^ SIL G/UV254 precoated plates (Macherey-Nagel; Düren, Germany). The spots were identified by using an UV lamp. For purification of products flash column chromatography was used with silica gel 40–63 μm from VWR International Chemicals (Darmstadt, Germany). The purity of all the tested compounds was >95% as determined by an LC-MS system including a diode array detector (DAD) detector (Dionex Ultimate 3000 system incorporating a LPG-3400SD pump, a WPS-3000 TSL autosampler, a TCC-3000SD column compartment, a DAD 3000 diode array detector and a MSQ 000 low resolution mass spectrometer (Thermo Fisher Scientific Inc.; Waltham, MA, USA), column: Reprosil-Pur Basic HD (150 × 3 mm; 3 µm; Dr. Maisch GmbH; Ammerbuch; Germany), gradient: 10-90-10% acetonitrile (MeCN)/20 mM NH_4_OAc_aq._ (*v*/*v*), run time: 15 min, flow rate: 0.6 mL/min, UV-detection: 254 nm). ^1^H-, ^13^C- and ^19^F-NMR spectra were recorded on VARIAN Mercury plus (300 MHz for ^1^H-NMR, 75 MHz for ^13^C-NMR, 282 MHz for ^19^F-NMR) and BRUKER DRX-400 (400 MHz for ^1^H-NMR, 100 MHz for ^13^C-NMR, 377 MHz for ^19^F-NMR); chemical shifts (*δ*) in parts per million (ppm) are related to internal tetramethylsilane and coupling constants (*J*) are given with 0.1 Hz (see Supplementary Materials, Figures S2−15). High resolution mass spectra (HRFT-MS) were recorded on a FT-ICR APEX II spectrometer (Bruker Daltonics; Bruker Corporation; Billerica, MA, USA) using electrospray ionization (ESI).

### 3.2. Chemical Synthesis

The corresponding fluoro benzoic acid **8** (1.1 eq, 0.21 mmol), (benzotriazol-1-yloxy)tris(dimethylamino)phosphonium hexafluorophosphate (BOP, 1.3 eq, 0.24 mmol) and triethylamine (3 eq, 0.56 mmol) were dissolved in dichlormethane (CH_2_Cl_2_, 3 mL) and stirred at room temperature (RT) for 30 min. After the addition of the 4-methoxy-7-morpholinobenzo[*d*]thiazol-2-amine 7 (50 mg, 1 eq, 0.19 mmol), the reaction mixture was stirred overnight at RT. The solvent was removed by rotatory evaporation and the remaining residue was dissolved in ethylacetate (EA, 10 mL). After the addition of 50 mL NaHCO_3_, the aqueous phase was extracted with EA (3 × 20 mL) and the combined organic phases were washed with brine (20 mL), dried over anhydrous Na_2_SO_4_, filtered and evaporated to dryness. The crude product was purified by flash chromatography (silica, gradient EA/PE 1:2 → 3:5 → 4:5 → 1:1) to afford the corresponding fluorinated derivative as a tan solid.

6-Fluoro-*N*-(4-methoxy-7-morpholinobenzo[*d*]thiazol-2-yl)nicotinamide (**TOZ1**). Yield: 81%; TLC (silica gel, CH_2_Cl_2_/MeOH, 9.5:0.5): R*f* = 0.55; ^1^H-NMR (300 MHz, DMSO-d_6_): *δ* = 13.26 (s, NH), 9.03−8.88 (m, 1H), 8.66 (ddd, *J* = 2.6, 7.7, 8.7 Hz, 1H), 7.41 (dd, *J* = 2.7, 8.6 Hz, 1H), 6.98 (d, *J* = 8.5 Hz, 1H), 6.94 (d, *J* = 8.5 Hz, 1H), 3.90 (s, 3H), 3.86−3.77 (m, 4H), 3.12−2.91 (m, 4H); ^19^F-NMR (282 MHz, DMSO-d_6_): *δ* = −63.35; ^13^C-NMR (101 MHz, DMSO-d_6_): *δ* = 166.07, 163.67, 156.92, 148.74 (d, *J* = 16.8 Hz), 148.28, 142.38 (d, *J* = 15.2 Hz), 140.08, 139.14, 126.65, 120.25, 113.25, 109.84 (d, *J* = 37.7 Hz), 108.22, 66.56, 55.98 (2C), 51.51 (2C); HRFT-MS (ESI+): m/z = 389.1086 (calcd. 389.1084 for [M+H]+).

2-Fluoro-*N*-(4-methoxy-7-morpholinobenzo[*d*]thiazol-2-yl)nicotinamide (**TOZ2**). Yield: 38%; TLC (silica gel, CH_2_Cl_2_/MeOH, 9.5:0.5): R_f_ = 0.55; ^1^H-NMR (300 MHz, DMSO-d_6_): *δ* = 13.13 (s, NH), 8.46 (ddd, *J* = 1.0, 2.0, 4.9 Hz, 1H), 8.38 (ddd, *J* = 2.0, 7.5, 9.5 Hz, 1H), 7.55 (ddd, *J* = 1.8, 4.9, 7.1 Hz, 1H), 6.99 (d, *J* = 8.6 Hz, 1H), 6.95 (d, *J* = 8.5 Hz, 1H), 3.89 (s, 3H), 3.83−3.72 (m, 4H), 3.10−2.93 (m, 4H); ^19^F-NMR (282 MHz, DMSO-d_6_): *δ* = 66.97 (d, *J* = 10.1 Hz); ^13^C-NMR (101 MHz, DMSO-d_6_): *δ* = 160.57, 158.18, 156.16, 150.67 (d, *J* = 15.0 Hz), 148.35, 141.92, 140.11, 139.00, 126.74, 122.27, 116.70 (d, *J* = 46.4 Hz), 116.47 (2C), 113.43, 108.40 (2C); HRFT-MS (ESI+): m/z = 389.1072 (calcd. 389.1084 for [M+H]+).

2-Fluoro-*N*-(4-methoxy-7-morpholinobenzo[*d*]thiazol-2-yl)isonicotinamide (**TOZ3**). Yield: 33%; TLC (silica gel, CH_2_Cl_2_/MeOH, 9.5:0.5): R_f_ = 0.55; ^1^H-NMR (300 MHz, DMSO-d_6_): *δ* = 13.42 (s, NH), 8.49 (dt, *J* = 0.8, 5.2 Hz, 1H), 7.99 (dt, *J* = 1.7, 5.2 Hz, 1H), 7.84 (d, *J* = 1.7 Hz, 1H), 7.00 (d, *J* = 8.6 Hz, 1H), 6.96 (d, *J* = 8.5 Hz, 1H), 3.90 (s, 3H), 3.87 − 3.72 (m, 4H), 3.12−2.95 (m, 4H); ^19^F-NMR (282 MHz, DMSO-d_6_): δ = -67.18; ^13^C-NMR (101 MHz, DMSO-d_6_): δ = 164.59, 162.34 (d, *J* = 118.5 Hz), 162.25, 148.8 (d, *J* = 55.6 Hz), 148.38, 145.06, 140.07, 136.29, 121.79, 120.41, 113.49, 108.72, 108.32, 66.55 (2C), 55.99, 51.51 (2C); HRFT-MS (ESI+): m/z = 389.1075 (calcd. 389.1084 for [M+H]+).

6-Fluoro-*N*-(4-methoxy-7-morpholinobenzo[*d*]thiazol-2-yl)picolinamide (**TOZ4**). Yield: 29%; TLC (silica gel, CH_2_Cl_2_/MeOH, 9.5:0.5): R_f_ = 0.55; ^1^H-NMR (400 MHz, DMSO-d_6_): *δ* = 12.40 (s, NH), 8.28 (q, *J* = 7.9 Hz, 1H), 8.12 (dd, *J* = 2.3, 7.4 Hz, 1H), 7.54 (d, *J* = 8.3 Hz, 1H), 7.12−6.80 (m, 2H), 3.91 (s, 3H), 3.81 (t, *J* = 4.6 Hz, 4H), 3.05 (t, *J* = 4.6 Hz, 4H); ^19^F-NMR (377 MHz, DMSO-d_6_): *δ* = −67.19; ^13^C-NMR (101 MHz, DMSO-d_6_): *δ* = 167.08, 163.44, 161.04, 148.87, 144.52 (d, *J* = 7.7 Hz), 144.25 (d, *J* = 8.0 Hz), 140.52, 123.73, 121.92, 113.92, 110.37, 110.01, 108.87, 67.01, 56.47, 55.35, 51.98 (2C); HRFT-MS (ESI+): m/z = 389.1071 (calcd. 389.1084 for [M+H]+).

4-Fluoro-*N*-(4-methoxy-7-morpholinobenzo[*d*]thiazol-2-yl)benzamide (**TOZ5**). Yield: 45%; TLC (silica gel, EA/PE, 1:1): R_f_ = 0.55; ^1^H-NMR (400 MHz, DMSO-d_6_): *δ* = 13.04 (s, NH), 8.24 (dd, *J* = 5.5, 8.7 Hz, 2H), 7.41 (t, *J* = 8.8 Hz, 2H), 6.97 (d, *J* = 8.5 Hz, 1H), 6.93 (d, *J* = 8.5 Hz, 1H), 3.89 (s, 3H), 3.85 − 3.76 (m, 4H), 3.09−2.99 (m, 4H); ^19^F-NMR (377 MHz, DMSO-d_6_): *δ* = −70.17 (d, *J* = 711.2 Hz); ^13^C-NMR (101 MHz, DMSO-d_6_): *δ* = 166.08, 163.58, 161.51, 148.23, 140.07, 133.13, 131.16 (d, *J* = 9.5 Hz, 2C), 128.28, 117.02, 115.76 (d, *J* = 22.0 Hz, 2C), 113.06, 108.12, 66.56 (2C), 55.93, 51.51 (2C); HRFT-MS (ESI+): m/z = 410.1009 (calcd. 410.0951 for [M+Na]+).

2-Fluoro-*N*-(4-methoxy-7-morpholinobenzo[*d*]thiazol-2-yl)benzamide (**TOZ6**). Yield: 86%; TLC (silica gel, EA/PE, 1:1): R_f_ = 0.55. ^1^H-NMR (400 MHz, DMSO-d_6_): *δ* = 12.95 (s, NH), 7.79 (ddd, *J* = 1.6, 5.1, 7.6 Hz, 1H), 7.66 (tdd, *J* = 1.8, 6.2, 8.4 Hz, 1H), 7.49−7.31 (m, 2H), 6.97 (d, *J* = 8.5 Hz, 1H), 6.94 (d, *J* = 8.5 Hz, 1H), 3.89 (s, 3H), 3.82−3.77 (m, 4H), 3.08−3.00 (m, 4H); ^19^F-NMR (377 MHz, DMSO-d_6_): *δ* = −70.16 (d, *J* = 711.3 Hz); ^13^C-NMR (101 MHz, DMSO-d_6_): *δ* = 163.78, 161.20, 157.76 (d, *J* = 188.1 Hz), 148.79, 140.56, 139.53, 134.48, 134.40, 130.82, 127.20, 125.12 (d, *J* = 3.3 Hz), 122.42 (d, *J* = 12.9 Hz), 116.92 (d, *J* = 21.5 Hz), 113.73, 108.77, 67.04 (2C), 56.46, 51.99 (2C); HRFT-MS (ESI+): m/z = 388.1189 (calcd. 388.1131 for [M+H]+).

4-Fluoro-*N*-(4-methoxy-7-morpholinobenzo[*d*]thiazol-2-yl)-3-nitrobenzamide (**TOZ7**). Yield: 42%; TLC (silica gel, DCM/MeOH, 9.5:0.5): R_f_ = 0.57; ^1^H NMR (300 MHz, DMSO-d_6_): *δ* = 13.35 (s, NH), 8.98 (dd, *J* = 2.3, 7.2 Hz, 1H), 8.54 (ddd, *J* = 2.4, 4.2, 8.8 Hz, 1H), 7.80 (dd, *J* = 8.7, 11.0 Hz, 1H), 6.99 (d, *J* = 8.6 Hz, 1H), 6.95 (d, *J* = 8.5 Hz, 1H), 3.91 (s, 3H), 3.81 (t, *J* = 4.6 Hz, 4H), 3.05 (t, *J* = 4.5 Hz, 4H); ^19^F-NMR (282 MHz, DMSO-d_6_): *δ* = −112.78; ^13^C-NMR (101 MHz, DMSO-d_6_): *δ* = 164.03, 160.29, 157.86 (d, *J* = 271.8 Hz), 147.31, 140.92, 137.03, 135.37 (d, *J* = 9.6 Hz), 129.86, 127.48, 126.61, 119.62 (d, *J* = 4.3 Hz), 119.15 (d, *J* = 21.5 Hz), 114.12, 107.42, 67.51 (2C), 55.54, 52.07 (2C); HRFT-MS (ESI+): m/z = 433.1045 (calcd. 433.0982 for [M+H]+).

*N*-(4-Methoxy-7-morpholinobenzo[*d*]thiazol-2-yl)-6-nitronicotinamide (**9**). A mixture of 6-fluoronicotinic acid (70 mg, 1.1 eq, 0.41 mmol), 1-[bis(di-methylamino)methylene]-1*H*-1,2,3-triazolo[4,5-*b*]pyridine-eium 3-oxide hexafluorophosphate (HATU, 186 mg, 1.3 eq, 0.49 mmol) and *N*,*N*-diisopropylethylamine (DIPEA, 198 µL, 3 eq, 1.13 mmol) in CH_2_Cl_2_ (3 mL) was stirred at RT for 30 min. After the addition of 4-methoxy-7-morpholinobenzo[*d*]thiazol-2-amine **7** (100 mg, 1 eq, 0.38 mmol) under argon atmosphere, the reaction mixture was stirred overnight at RT and then diluted with water (20 mL). The aqueous phase was extracted with CH_2_Cl_2_ (3 × 20 mL) and the combined organic phases were washed with NaHCO_3_ (20 mL) and brine (20 mL), dried over anhydrous MgSO_4_, filtered and evaporated to dryness. The crude product was purified by flash chromatography (silica, gradient CH_2_Cl_2_/MeOH 100:1 → 100:2 → 100:3) to give **9** (47 mg, 0.14 mmol, 36%) as a yellow solid. TLC (silica gel, CH_2_Cl_2_/MeOH, 9.5:0.5): R_f_ = 0.56; ^1^H-NMR (400 MHz, DMSO-d_6_): *δ* = 9.29 (d, *J* = 2.2 Hz, 1H, 8), 8.83 (d, *J* = 8.4 Hz, 1H, 9), 8.48 (d, *J* = 8.4 Hz, 1H, 10), 7.15 − 6.80 (m, 2H, 1/2), 3.91 (s, 3H, 7), 3.80 (t, *J* = 4.5 Hz, 4H, 5/6), 3.13-2.90 (m, 4H, 3/4); ^13^C-NMR (101 MHz, DMSO-d_6_): *δ* = 164.26, 159.24, 158.37, 149.45, 48.55, 141.30, 141.03, 140.87, 134.75, 127.35, 118.32, 113.77, 109.69, 67.10 (2C), 56.85, 52.07 (2C).

### 3.3. Docking Stimulation

Molecular docking studies were carried out using the Genetic Optimization for Ligand Docking (GOLD) 5.5 program from Cambridge Crystallographic Data Center (CCDC; Cambridge; UK). GOLD uses a genetic algorithm for docking ligands into protein binding sites to explore the full range of ligand conformational flexibility with partial flexibility of the active site of the protein. The X-ray crystallographic chimeric protein structure of A_2A_R–BRIL in complex with the antagonist **ZM241385** (PDB ID: 4EIY) was considered for the purpose of docking stimulation. Among the several other crystal structures in the Protein Data Bank (PDB), this structure was particularly selected due to it having the highest resolution of all available non-thermostabilized structures. The A_2A_R protein was prepared by using the protein preparation wizard tool implemented in the GOLD software that removes all water molecules and adds hydrogen atoms to the protein structure. After the definition of the active site with a 4 Å radius around the ligand Z**M241385** present in the orthosteric binding site of the A_2A_R, **ZM241385** was removed from the protein structure. The ligand preparation was carried out in the CambridgeSoft Chem3D 17.0 program from PerkinElmer (USA). The energy of each compound was minimized by using the MM2 force field method. Ten docking runs were performed per structure and the early termination step was activated if the first three poses have a root-mean-square deviation (RMSD) value of less than 1.5 Å, other parameters were set as default. After docking, the individual binding poses of each compound were observed and their molecular interactions within the active site were evaluated. The program Discovery Studio 2017 from BIOVIA^®^ (San Diego, CA, USA) was used to visualize the key aspects of the docking results from GOLD.

### 3.4. Manual Radiosynthesis

No carrier added (n.c.a.) [^18^F]fluoride was produced via the [^18^O(p,n)^18^F] nuclear reaction by irradiation of an [^18^O]H2O target (Hyox 18 enriched water; Rotem Industries Ltd.; Mishor Yamin, Israel) on a Cyclone 18/9 (iba RadioPharma Solutions; Louvain-la-Neuve, Belgium) with a fixed energy proton beam using Nirta [^18^F]fluoride XL target. N.c.a. [^18^F]fluoride in 1 mL of water was trapped on a pre-conditioned Sep-Pak Accell Plus QMA Carbonate Plus light cartridge (Waters GmbH; Eschborn, Germany, pre-conditioned with 15 mL 0.5 M NaHCO_3_ and 10 mL H_2_O). Then, the activity was eluted with 300 μL of an aqueous K_2_CO_3_ solution (20 mg/mL solution, 0.9 mg, 6.5 μmol) in a conical 4 mL vial with kryptofix^®^ (K_222_, 5.6 mg, 14.9 μmol) in 1 mL of MeCN. The aqueous [^18^F]fluoride was azeotropically dried under vacuum and nitrogen flow within 7–10 min using a CEM Discover PETwave Microwave (CEM GmbH; Kamp-Lintfort, Germany; 75 W, 50–60 °C, power cycling mode). Two aliquots of anhydrous MeCN (2 × 1.0 mL) were added during the drying procedure and, then, the final K[^18^F]F–K_222_ complex was dissolved in 0.2 mL DMSO. The K[^18^F]F–K_222_ complex was stirred with the nitro precursor **9** (0.5 mg in 0.4 mL DMSO) at 180 °C for 15 min. After cooling, the reaction mixture was diluted with 0.4 mL MeCN and 3 mL water and injected onto a semi-preparative HPLC (ReproSil-Pur 120 C18-AQ column (250 × 10 mm, particle size: 5 µm), 36% MeCN/ H_2_O/0.05% trifluoroacetic acid (TFA), flow rate: 4 mL/min). The fractions containing [^18^F]**TOZ1** were collected and diluted with water to a total volume of 40 mL. Thereafter, the solution was passed through a pre-conditioned Sep Pak^®^ C18 light cartridge (Waters GmbH; Eschborn, Germany; pre-conditioned with 5 mL EtOH and 60 mL H_2_O), washed with 2 mL water, and [^18^F]**TOZ1** subsequently eluted with 1 mL absolute EtOH. The solvent was reduced under a stream of nitrogen at 70 °C (approx. 20 μL), and [^18^F]**TOZ1** was formulated with the addition of sterile isotonic saline solution up to a final concentration of <10% EtOH (*v*/*v*). Radiochemical and chemical purities were assessed by radio-TLC and analytical HPLC. Molar activities were determined based on aliquots taken from the formulation, and the mass determination for the corresponding reference standard was performed via a calibration curve (0.05–20 µg **TOZ1**) obtained under the same analytical HPLC conditions (see quality control section).

### 3.5. Automated Radiosynthesis

Remote controlled automated synthesis was performed using a raytest SynChrom R&D synthesis module from Elysia-Raytest GmbH (Staubenhard, Germany). N.c.a. [^18^F]fluoride was produced as previously described. The K[^18^F]F–K_222_ complex was obtained after trapping [^18^F]fluoride on a pre-conditioned Sep-Pak Accell Plus QMA Carbonate Plus light cartridge (Waters GmbH; Eschborn, Germany, pre-conditioned with 15 mL 0.5 M NaHCO_3_ and 10 mL H_2_O), elution with a solution containing K_2_CO_3_ (0.9 mg in 0.3 mL H_2_O) and K_222_ (5.6 mg in 0.8 mL MeCN) and azeotropically distillation. Then, the K[^18^F]F–K_222_ complex was stirred with the nitro precursor **9** (0.5 mg in 0.6 mL DMSO) at 180 °C for 15 min. After cooling, the reaction mixture was diluted with 0.4 mL MeCN and 3 mL water, transferred into the second reaction vessel and injected onto a semi-preparative HPLC (ReproSil-Pur 120 C18-AQ column (250 × 10 mm, particle size: 5 µm), 36% MeCN/H_2_O/0.05% TFA, flow rate: 4 mL/min). The fractions containing [^18^F]**TOZ1** were transferred to a collection vial that was previously loaded with 30 mL water. Thereafter, the solution was passed through a pre-conditioned Sep Pak^®^ C18 light cartridge (Waters GmbH; Eschborn, Germany; pre-conditioned with 5 mL EtOH and 60 mL H_2_O), washed with 2 mL water; and [^18^F]**TOZ1** was subsequently eluted with 1.2 mL absolute EtOH into the product vial. Then, the ethanolic solution was transferred out of the hot cell and the solvent was reduced under a stream of nitrogen at 70 °C (approx. 20 μL), and [^18^F]**TOZ1** was formulated with the addition of sterile isotonic saline solution up to a final concentration of <10% EtOH (*v*/*v*). Radiochemical and chemical purities were assessed by radio-TLC and analytical HPLC. Molar activities were determined based on aliquots taken from the formulation, and the mass determination for the corresponding reference standard was performed via a calibration curve obtained under the same analytical HPLC conditions (see quality control section).

### 3.6. Quality Control

Radio-TLC was performed on silica gel (Polygram^®^ SIL G/UV_254_; Roth; Germany) pre-coated plates with a mixture of CH_2_Cl_2_/MeOH 9.5/0.5 (*v*/*v*) as eluent. The plates were exposed to storage phosphor screens (BAS-IP MS 2025; FUJIFILM Co.; Tokyo; Japan) and recorded using the Amersham Typhoon RGB Biomolecular Imager (GE Healthcare Life Sciences). Images were quantified with the ImageQuant TL8.1 software (GE Healthcare Life Sciences). HPLC analysis were performed on a JASCO LC-2000 system, incorporating a PU-2080Plus pump, AS-2055Plus auto injector (100 μL sample loop), and a UV-2070Plus detector coupled with a gamma radioactivity HPLC detector (Gabi Star; raytest Isotopenmessgeräte GmbH; Staubenhardt, Germany) and RP-HPLC columns from Dr. Maisch HPLC GmbH (Ammerbruch, Germany). Data analysis was performed with the Galaxie chromatography software (Agilent Technologies): ReproSil-Pur 120 C18-AQ column (250 × 4.6 mm, particle size 5 µm), eluent: MeCN/ 20 mM NH_4_OAc_aq._, gradient mode (0–5 min 10% MeCN, 5–30 min up to 90% MeCN, 30–35 min 90% MeCN, 35–40 min up to 10% MeCN, 40–45 min 10% MeCN), flow rate: 1 mL/min, UV-detection: 254 nm. The molar activities were determined on the basis of a calibration curve (0.05–20 µg **TOZ1**) carried out under isocratic HPLC conditions (36% MeCN/H_2_O/0.05% TFA, analytical ReproSil-Pur 120 C18-AQ column) using chromatograms obtained at 244 nm as the maximum of UV absorbance.

### 3.7. In Vitro stability And Lipophilicity (LogD_7.4_)

The chemical stability of [^18^F]**TOZ1** was investigated in isotonic saline, phosphate-buffered saline (PBS, pH 7.4) and *n*-octanol by incubation at 37 °C. Samples were taken at 15, 30 and 60 min of incubation time and analyzed by radio-TLC and radio-HPLC. The log*D*_7.4_ value of [^18^F]**TOZ1** was experimentally determined in *n*-octanol/PBS at RT by the shake-flask method [46]. The measurements were performed in triplicate.

### 3.8. Biological Evaluation

All experimental work including the use of animals was conducted in accordance with the national legislation on the use of animals for research (Tierschutzgesetz (TierSchG), Tierschutz-Versuchstierverordnung (TierSchVersV)) and were approved by the Animal Care and Use Committee of Saxony (TVV 18/18 Landesdirektion Sachsen). All animal experiments were performed with female CD-1 mice (10–12 weeks, 26–38 g) obtained from the Medizinisch-Experimentelles Zentrum (MEZ) at Universität Leipzig (Leipzig, Germany).

### 3.9. In Vitro Binding Assays

CHO-K1 cells stably transfected with the human A_1_R or A_2A_R, a donation from Prof. Karl-Norbert Klotz (Institute of Pharmacology and Toxicology; Universität Würzburg; Würzburg, Germany), were cultured in DMEM/F12 medium supplemented with 15 mM HEPES, 10% FCS, 1% L-Glutamine, 1% Penicillin/Streptomycin, and G418 as selective antibody at 0.2 mg/mL at 5% CO_2_ and 37 °C. Cells were harvested by scraping followed by centrifugation (800 rpm, 5 min), and the resulting pellet incubated in 50 mM TRIS-HCl, ph 7.4, on ice for 20 min. The crude membrane homogenate was obtained by centrifugation (15,000 rpm; 30 min, 4 °C), suspended in 50 mM TRIS-HCl, pH 7.4, and stored at −25 °C. Membrane suspension was incubated with the A_2A_R-specific [^3^H]**ZM241385** (American Radiolabelled Chemicals Inc.; ART0884; A_m_ = 1.851 TBq/mmol) (*K*_i_(*h*A_2A_R) = 0.8 nM); or the A_1_-specific [^3^H]**DPCPX** (PerkinElmer; NET974250UC, A_m_ = 6.068 TBq/mmol) (*K*_i_(*h*A_1_R) = 0.45 nM), and the test compound at different concentrations in buffer at RT. Non-specific binding was determined by co-incubation with 10 μM **ZM241385** or 1 μM **DPCPX**. The IC_50_ values were determined by non-linear regression analysis with GraphPad Prism 4.1 (GraphPad Inc., La Jolla, CA, USA), and K_i_ values were estimated according to the Cheng-Prusoff equation with *K*_D,ZM241385_(*h*A_2A_R) = 0.8 nM and *K*_D,DPCPX_(*h*A_1_R) = 0.45 nM.

### 3.10. In Vitro Autoradiography

Brains of CD-1 mice and piglets frozen in isopentane were cut using a cryostat, thaw-mounted onto microscope slides, and after air-drying stored at −80 °C until use. Briefly, the brain cryosections were dried in a stream of cold air, and pre-incubated in 50 mM TRIS HCl buffer (pH 7.4, 100 mM NaCl, 5 mM MgCl2, 1 mM EDTA) containing 1 µU/mL adenosine deaminase (ADA) for 15 min at RT. Afterwards, brain sections were incubated with 0.1 MBq/mL of [^18^F]**TOZ1** (1.1 nM) in buffer for 90 min at RT. Non-specific binding was determined in the presence of 1 µM **ZM241385**. Displacement was evaluated with different concentrations of **TOZ1** in order to determine the binding affinity towards the A_2A_R. Subsequently, the sections were washed twice for 5 min in ice-cold TRIS-HCl buffer and dipped for 5 s in ice-cold deionized water. The sections were rapidly dried in a stream of cold air before being exposed overnight on an imaging plate. Developed autoradiographs were analyzed in a phosphor imager (HD-CR 35; Duerr NDT GmbH; Bietigheim-Bissingen, Germany). Quantification was performed by using 2D-densitometric analysis (AIDA 2.31 software; raytest Isotopenmessgeräte GmbH; Straubenhardt, Germany). Further data analysis was performed with GraphPad Prism 4.1 (GraphPad Inc.; La Jolla, CA, USA).

### 3.11. In Vivo Metabolism

The radiotracer was administered i.v. as bolus in awake CD-1 mice (40 MBq [^18^F]**TOZ1**; n = 2). At 30 min post injection (p.i.), blood samples were taken retroorbitally from the anesthetized animals. Plasma was separated by centrifugation at 8000 rpm at RT for 1 min (Centrifuge 5418; Eppendorf Vertrieb Deutschland GmbH; Wesseling-Berzdorf; Germany), and brain homogenized in 1 mL water on ice (10 strokes of a PTFE plunge at 1000 rpm in a borosilicate glass cylinder; Potter S Homogenizer; B. Braun Melsungen AG; Melsungen, Germany). For protein precipitation and extraction an ice-cold mixture of acetone/water (4/1; *v*/*v*) was used in a ratio of 4:1 (*v*/*v*) of solvent to plasma or brain homogenate, respectively. The samples were vortexed for 3 min, equilibrated on ice for 5 min, and centrifuged for 5 min at 10,000 rpm. After separating the supernatant, the precipitates were washed with 100 µL of the solvent mixture and subjected to the same procedure. The combined supernatants were concentrated at 75 °C under nitrogen flow to a final volume of approx. 100 µL and analyzed by analytical radio-HPLC (see section quality control). To determine the percentage of activity in the supernatants compared to total activity, aliquots of each step as well as the precipitates were quantified by a gamma counter (Wallac Wizard 1480; Perkin Elmer; Turku; Finland).

### 3.12. PET Imaging

For the time of the experiments, CD-1 mice were kept in a dedicated climatic chamber with free access to water and food under a 12:12 h dark:light cycle at a constant temperature (24 °C). The animals were anaesthetized (anesthesia unit U-410; agntho’s; Lidingö; Sweden) with isoflurane (1.8%, 0.35 L/min) delivered in a 40% oxygen/60% air mixture (Gas Blender 100 Series; MCQ instruments; Rome, Italy) and maintained at 37 °C with a thermal bed system. The formulated radiotracer was injected into the tail vein ([^18^F]**TOZ1**: 5.3 ± 8.3 MBq in 150 µL isotonic saline; A_m_: 66 GBq/µmol; 7.9 ± 5.8 nmol/kg) followed by a 60 min PET/MR scan (PET/MR 1Tesla; nanoScan^®^; MEDISO Medical Imaging Systems; Budapest, Hungary): 7 baseline studies with vehicle (DMSO/Kolliphor/NaCl, 1:2:7, *v*/*v*/v), 3 pre-treatment studies with **tozadenant** (2.5 mg/kg; abcr GmbH; Karlsruhe, Germany) and 4 pre-treatment studies with **cyclosporine A** (50 mg/kg; Sigma Aldrich, Germany). Each PET image was corrected for random coincidences, dead time, scatter and attenuation (AC), based on a whole body (WB) MR scan. The list mode data were sorted into sinograms using a framing scheme of 12 × 10 s, 6 × 30 s, 5 × 300 s, 9 × 600 s. The reconstruction parameters for the list mode data were 3D-ordered subset expectation maximization (OSEM), 4 iterations, 6 subsets, energy window: 400–600 keV, coincidence mode: 1–5, ring difference: 81. The mice were positioned prone in a special mouse bed (heated up to 37 °C), with the head fixed to a mouth piece for the anesthetic gas supply with isoflurane in 60% air and 40% oxygen (anesthesia unit: U-410, agnthos, Lidingö, Sweden; Gas blender: MCQ, Rome, Italy). The PET data were collected by a continuous WB scan during the entire investigation. Following the 60 min PET scan a T1 weighted WB gradient echo sequence (TR/TE: 20/6.4 ms, NEX: 1, FA: 25, FOV: 64 × 64 mm, matrix: 128 × 128, slice thickness: 0.5 mm) was performed for AC and anatomical orientation. Image registration and evaluation of the volumes of interest (VOI) was done with PMOD 3.9 (PMOD technologies LLC; Zurich, Switzerland). The respective brain regions were identified using the mouse brain atlas template Ma-Benveniste-Mirrione-FDG. Spherical VOI with diameters of 1 to 2 mm were placed at the center of the liver, stomach and spleen. Gallbladder, small intestine, kidney, and bladder VOI were delineated from the PET signal. Heart wall, stomach wall and blood were delineated from the T1 weighted image avoiding spill over from neighboring organs. The activity data are expressed as mean SUV + SEM of the overall VOI.

## 4. Conclusions

We herein described a series of novel fluorinated derivatives (**TOZ1**-**TOZ7**) based on chemical modification of the clinically relevant A_2A_R antagonist **tozadenant**. Among those, the highly affine **TOZ1** was selected for the development of an ^18^F-labeled PET radiotracer for A_2A_R imaging in the brain. A fully automated radiosynthesis of [^18^F]**TOZ1** was successfully established via radiofluorination of the corresponding nitro precursor. The target-specific binding of [^18^F]**TOZ1** was demonstrated by autoradiography in mouse and piglet brain. Metabolism studies in mice revealed a high in vivo stability with only one minor brain-penetrating radiometabolite. While dynamic PET studies in mice revealed penetration of the BBB of [^18^F]**TOZ1**, target specific binding was insufficient. Our ongoing work is focusing on further medicinal chemistry of other potential lead compounds to develop suitable A_2A_R PET radiotracers.

## Figures and Tables

**Table 1:** Overview of synthesized **TOZ** derivatives and in-house determined in vitro binding of Figure 2.

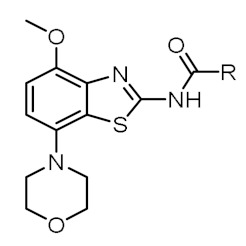	R	*K*_i_(*h*A_2A_R) in nM	*K*_i_(*h*A_1_R) in nM	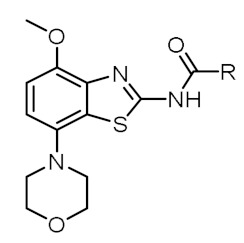	R	*K*_i_(*h*A_2A_R) in nM	*K*_i_(*h*A_1_R) in nM
**Tozadenant**	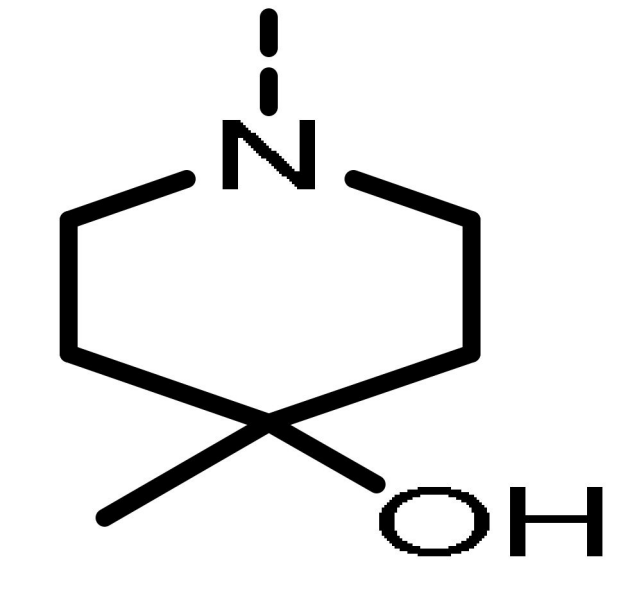	2.39 (2.04, 2.74)	897 (841, 952)	**TOZ4**	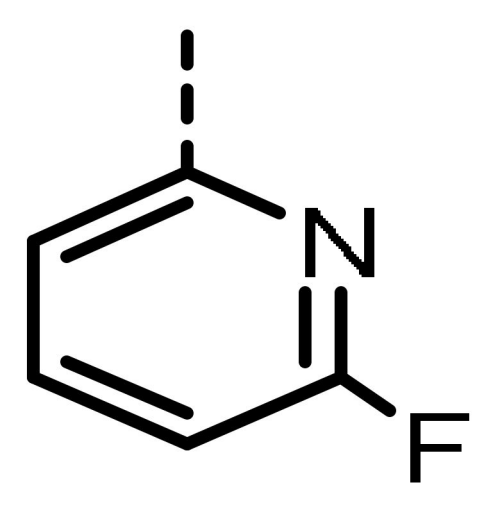	n.d.	n.d.
**TOZ1**	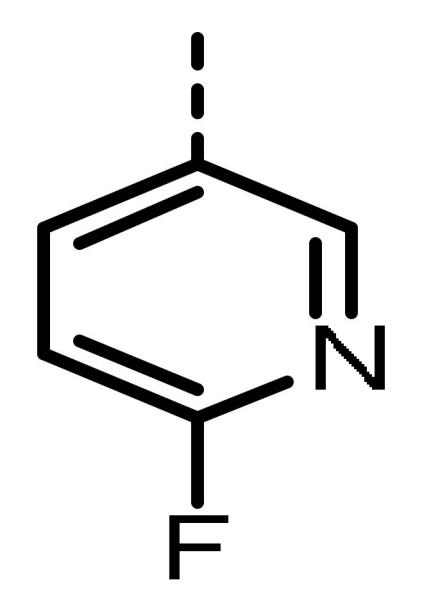	1.00 ± 0.16	618 ± 31	**TOZ5 (6a)**	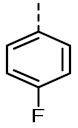	1.04 (1.09, 1.03)	336 (376, 271)
**TOZ2**	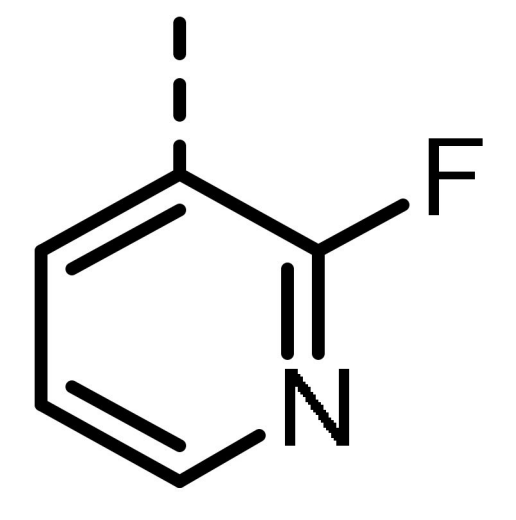	5.02 (5.57, 4.78)	1398 (1547, 1294)	**TOZ6**	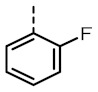	4.84 (4.96, 4.74)	239 (488, 132)
**TOZ3**	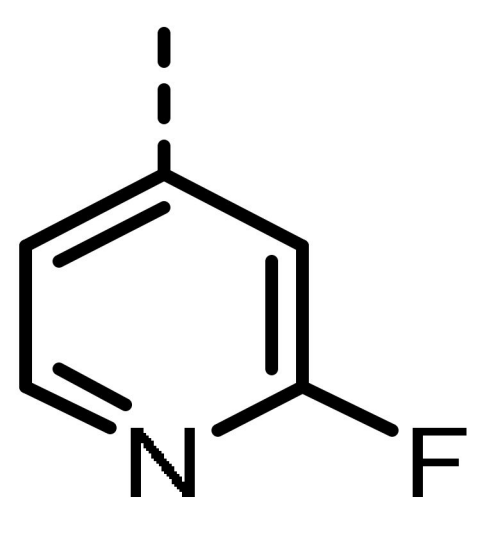	2.56 ± 0.11	729 ± 22	**TOZ7**	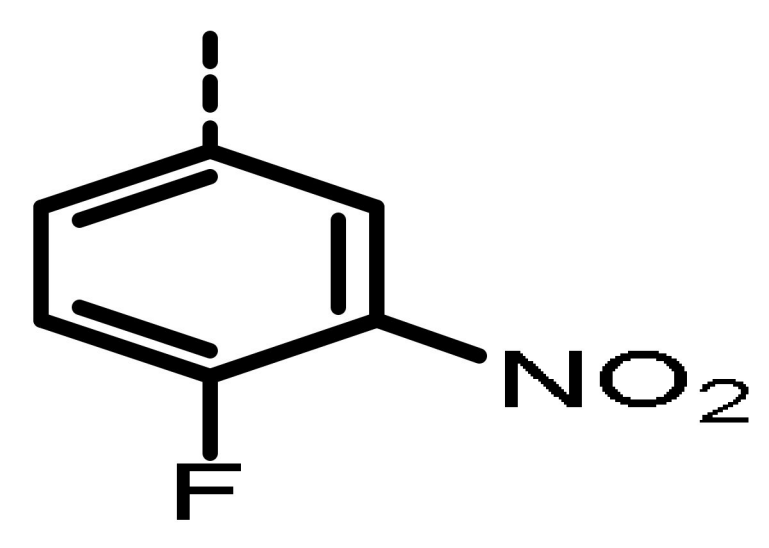	3.23 (3.47, 2.99)	4278 (5069, 3487)

A_2A_R and A_1_R subtypes; mean value ± standard deviation (*n* ≥ 3) or mean value (single values, *n* = 2); n.d. = not determined.

## Data Availability

The data that support the findings of this study are available from the corresponding author upon reasonable request.

## Supplementary Materials

The following are available online at https://www.mdpi.com/1422-0067/22/3/1182/s1, Figure S1: Scheme of the raytest SynChrom R&D synthesis module for the automated radiosynthesis of [^18^F]**TOZ1**. Figures S2–S15: ^1^H-NMR and LC-MS of **TOZ1**-**TOZ7**.
