# Supplementary material for: Synthesis and Biological Evaluation of a Novel 18F-Labeled Radiotracer for PET Imaging of the Adenosine A2A Receptor
Source: Int J Mol Sci. 2021 Jan 25;22(3):1182. doi: 10.3390/ijms22031182 (PMC7865263; doi:10.3390/ijms22031182)
Supplement: Supplementary file 1 [file ijms-22-01182-s001.pdf]

International Journal of *Molecular Sciences*

MDPI

Supplementary Materials

Synthesis and Biological Evaluation of a Novel ^{18}F-Labeled Radiotracer for PET Imaging of the Adenosine A_{2A} Receptor

Thu Hang Lai [1,2,*,†], Magali Toussaint [1,*,†], Rodrigo Teodoro [1,†], Sladjana Dukić-Stefanović [1], Mathias Kranz [1,3,4], Winnie Deuther-Conrad [1], Rareş-Petru Moldovan [1] and Peter Brust [1]

1 Helmholtz-Zentrum Dresden-Rossendorf (HZDR), Department of Neuroradiopharmaceuticals, Institute of Radiopharmaceutical Cancer Research, Research site Leipzig, 04318 Leipzig, Germany; r.teodoro@hzdr.de (R.T.); s.dukic-stefanovic@hzdr.de (S.D.-S.); mathias.kranz@uit.no (M.K.); w.deuther-conrad@hzdr.de (W.D.-C.); r.moldovan@hzdr.de (R.-P.M.); p.brust@hzdr.de (P.B.)

2 Department of Research and Development, ROTOP Pharmaka Ltd., 01328 Dresden, Germany

3 PET Imaging Center, University Hospital of North Norway (UNN), 9009 Tromsø, Norway

4 Nuclear Medicine and Radiation Biology Research Group, The Arctic University of Norway, 9009 Tromsø, Norway

* Correspondence: t.lai@hzdr.de (T.H.L.); m.toussaint@hzdr.de (M.T.); Tel.: +49-341-234-179-4635 (T.H.L.); +49-341-234-179-4616 (M.T.)

† These authors contributed equally to the work.

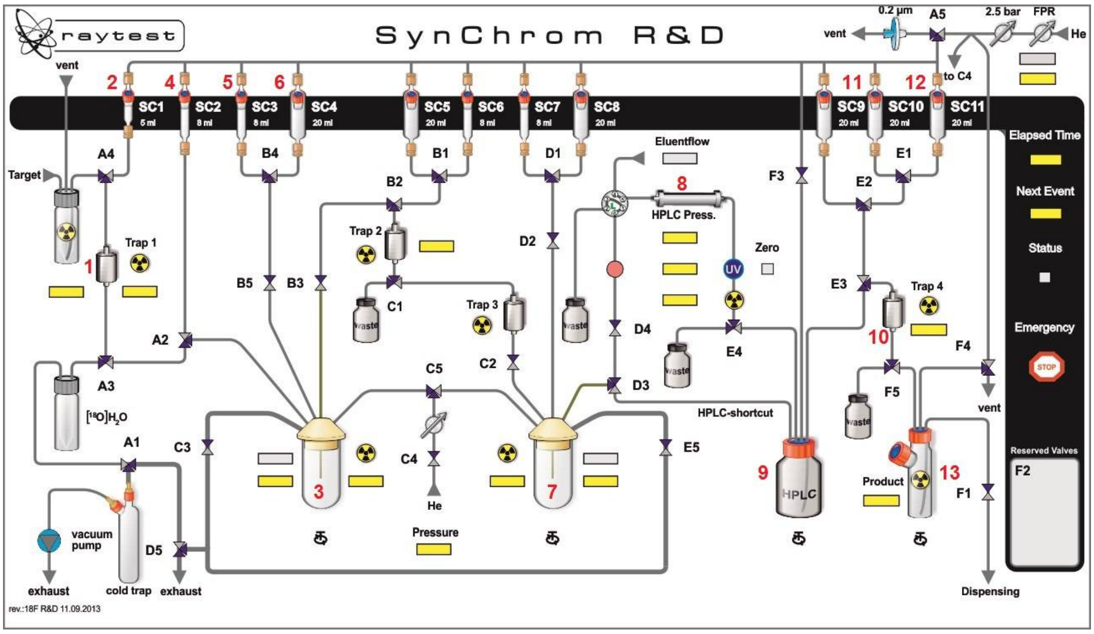

Figure S1: Scheme of the *raytest SynChrom* R&D synthesis module for the automated radiosynthesis of [^{18}F]**TOZ1** with (**1**) Sep-Pak® Accell Plus QMA Plus Light catridge, (**2**) K_2CO_3 (0.9 mg in 0.2 mL H_2O) and K_{222} (5.6 mg in 0.8 mL MeCN), (**3**) 1st reactor, (**4**) 1.5 mL MeCN, (**5**) precursor **9** (0.5 mg in 0.6 mL DMSO), (**6**) 0.4 mL MeCN and 3 mL H_2O, (**7**) 2nd reactor, (**8**) Reprosil-Pur C18-AQ column (36% MeCN/H_2O/0.05% TFA, flow rate of 4 mL/min), (**9**) 30 mL water, (**10**) Sep-Pak® C18 light cartridge, (**11**) 2 mL H_2O, (**12**) 1.2 mL EtOH, (13) product vial.

6-Fluoro-*N*-(4-methoxy-7-morpholinobenzo[*d*]thiazol-2-yl)nicotinamide (TOZ1)

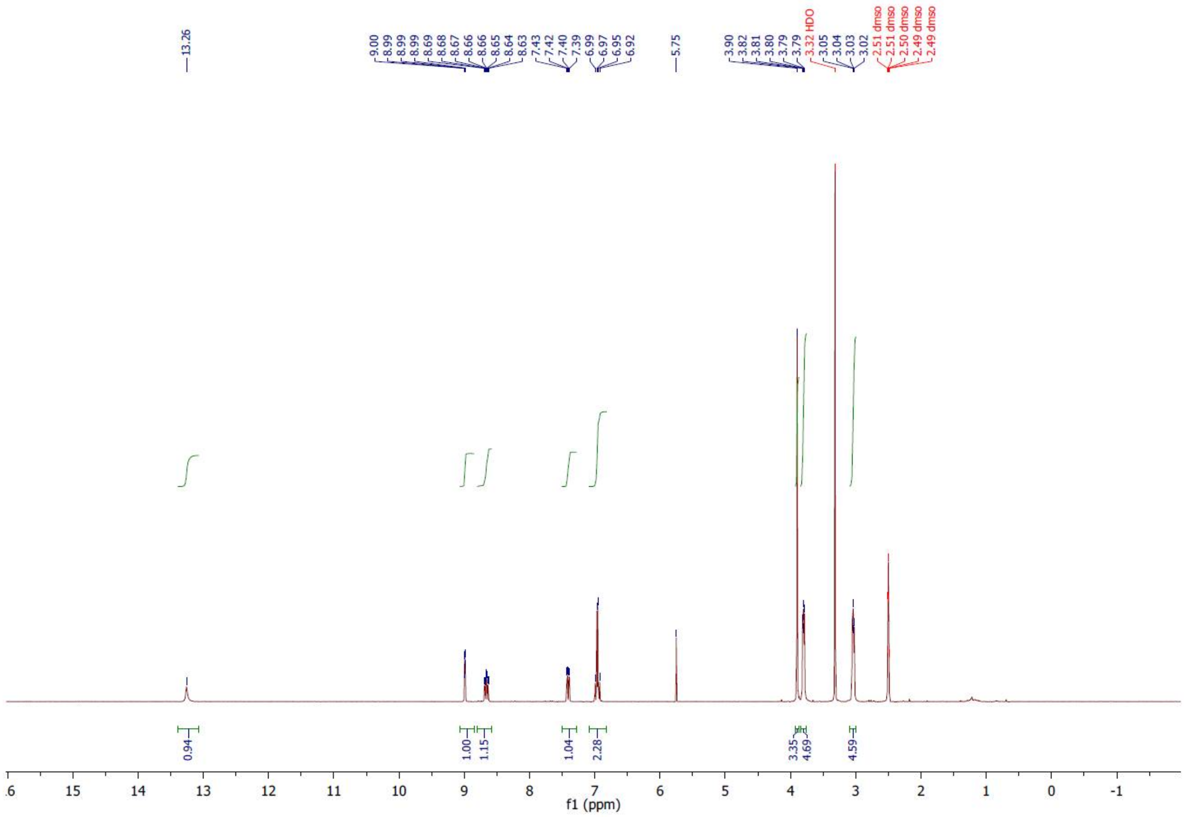

Figure S2: ^{1}H-NMR of **TOZ1**.

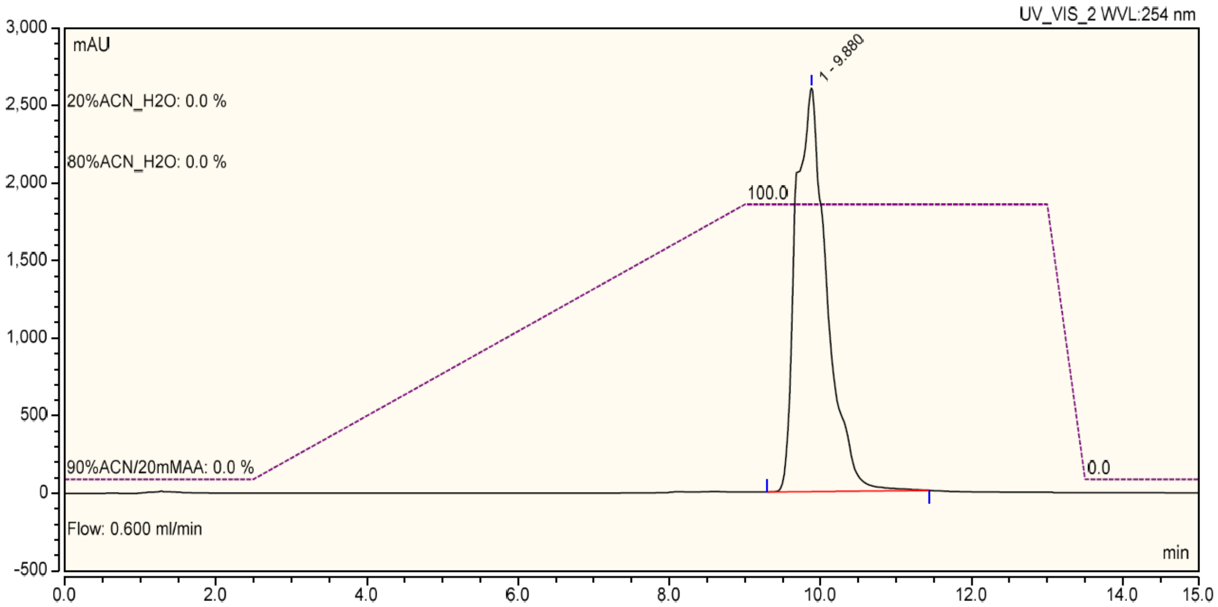

Figure S3: LC-MS chromatogram of **TOZ1**.

2-Fluoro-*N*-(4-methoxy-7-morpholinobenzo[*d*]thiazol-2-yl)nicotinamide (TOZ2)

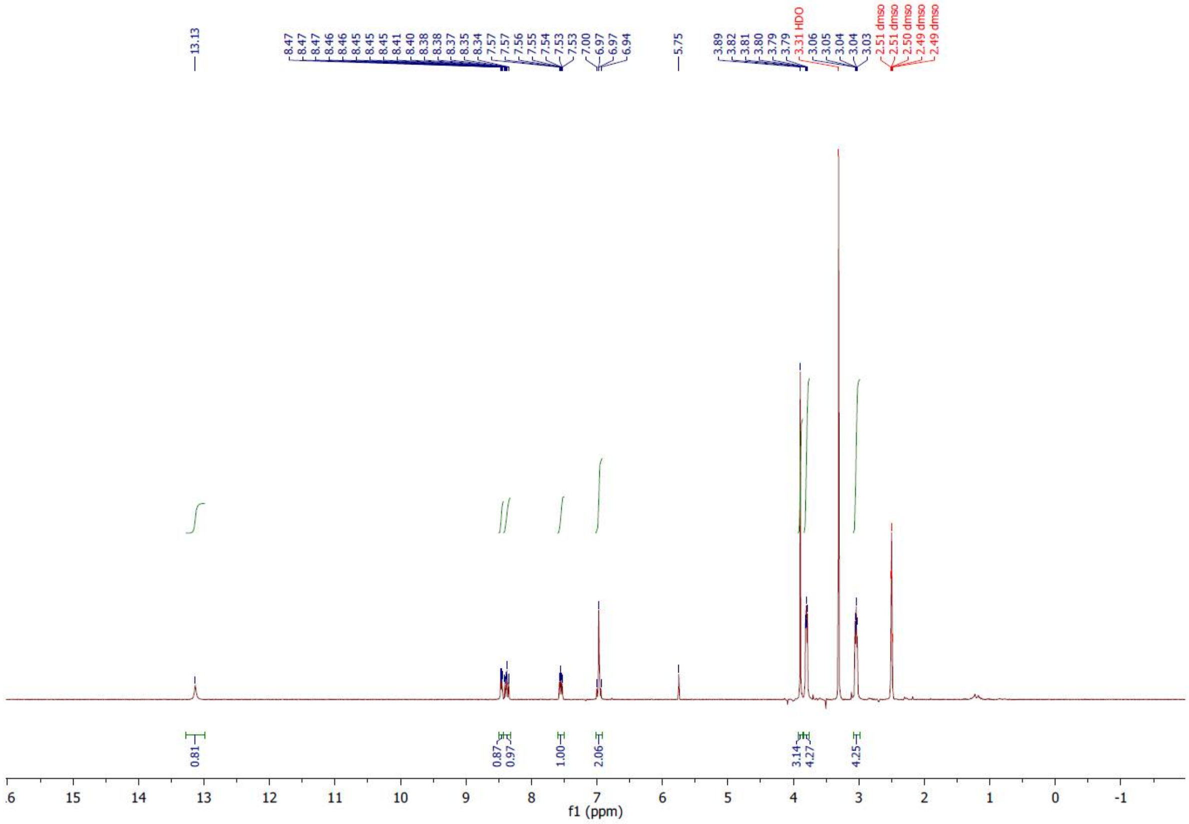

Figure S4: ^{1}H-NMR of **TOZ2**.

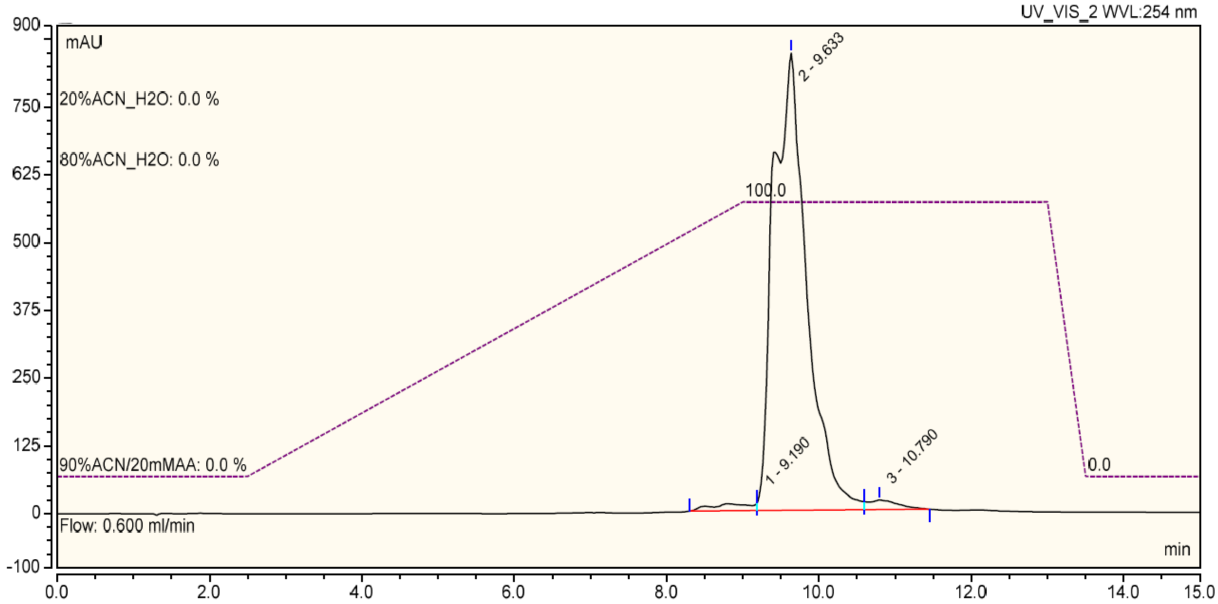

Figure S5: LC-MS chromatogram of **TOZ2**.

2-Fluoro-*N*-(4-methoxy-7-morpholinobenzo[*d*]thiazol-2-yl)isonicotinamide (TOZ3)

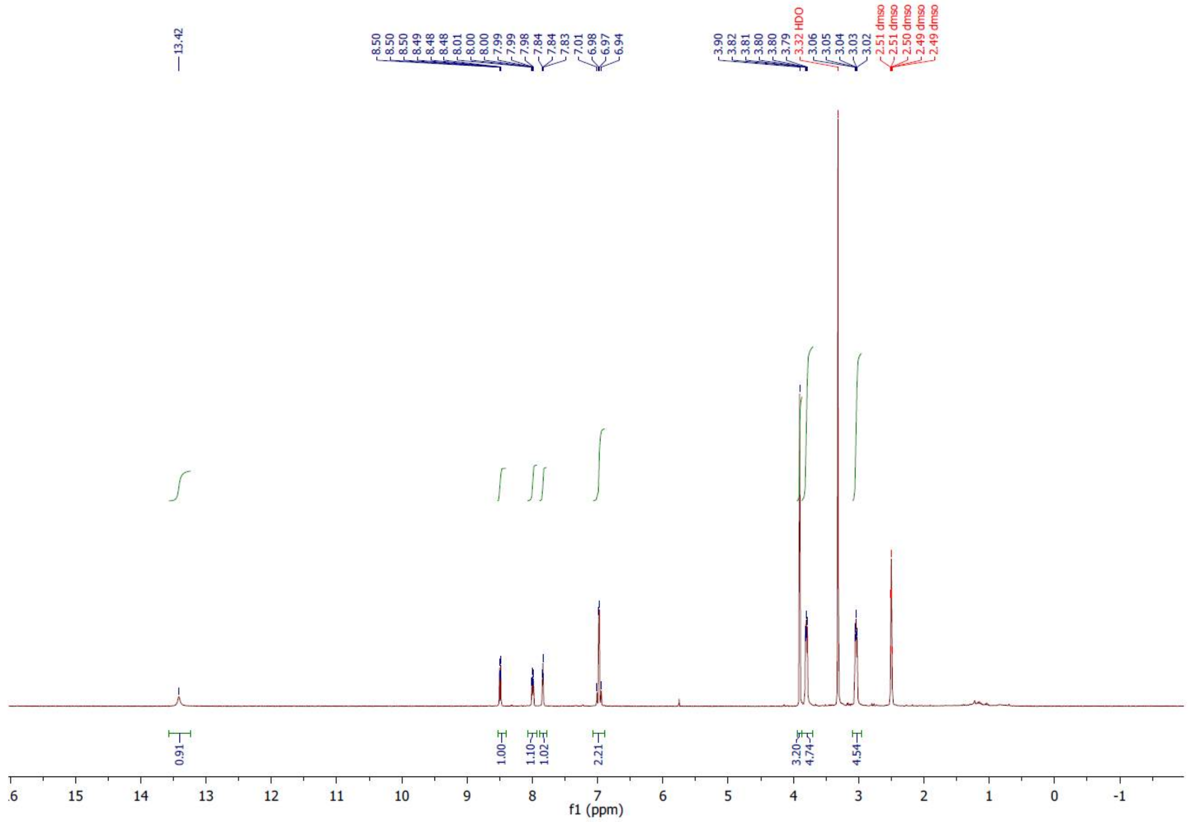

Figure S6: ^{1}H-NMR of **TOZ3**.

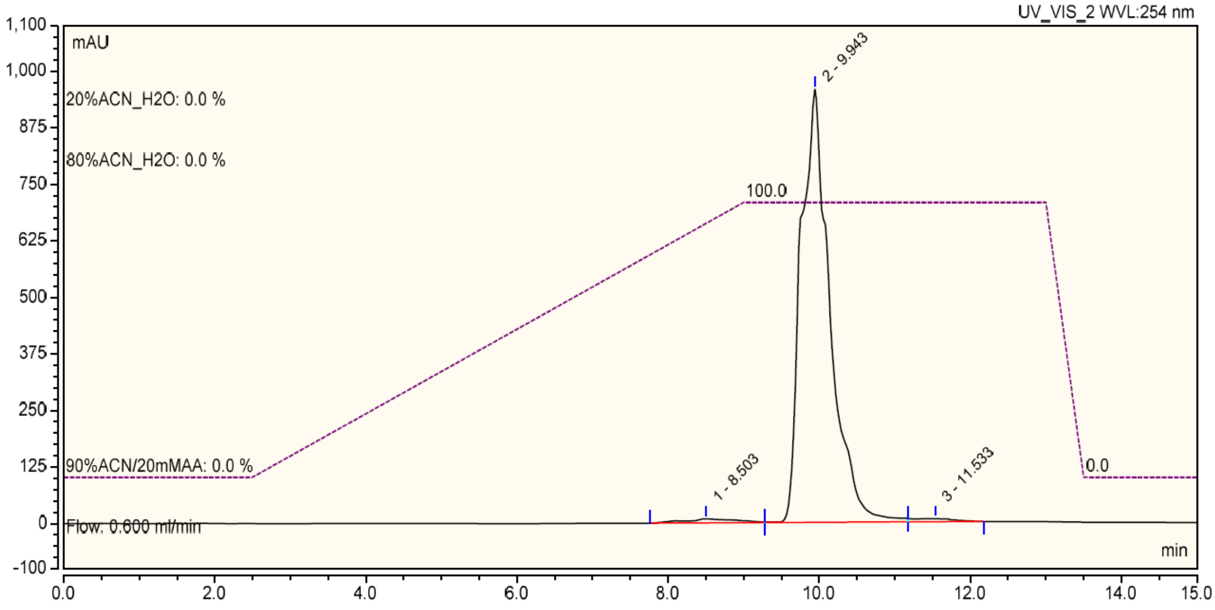

Figure S7: LC-MS chromatogram of **TOZ3**.

6-Fluoro-*N*-(4-methoxy-7-morpholinobenzo[*d*]thiazol-2-yl)picolinamide (TOZ4)

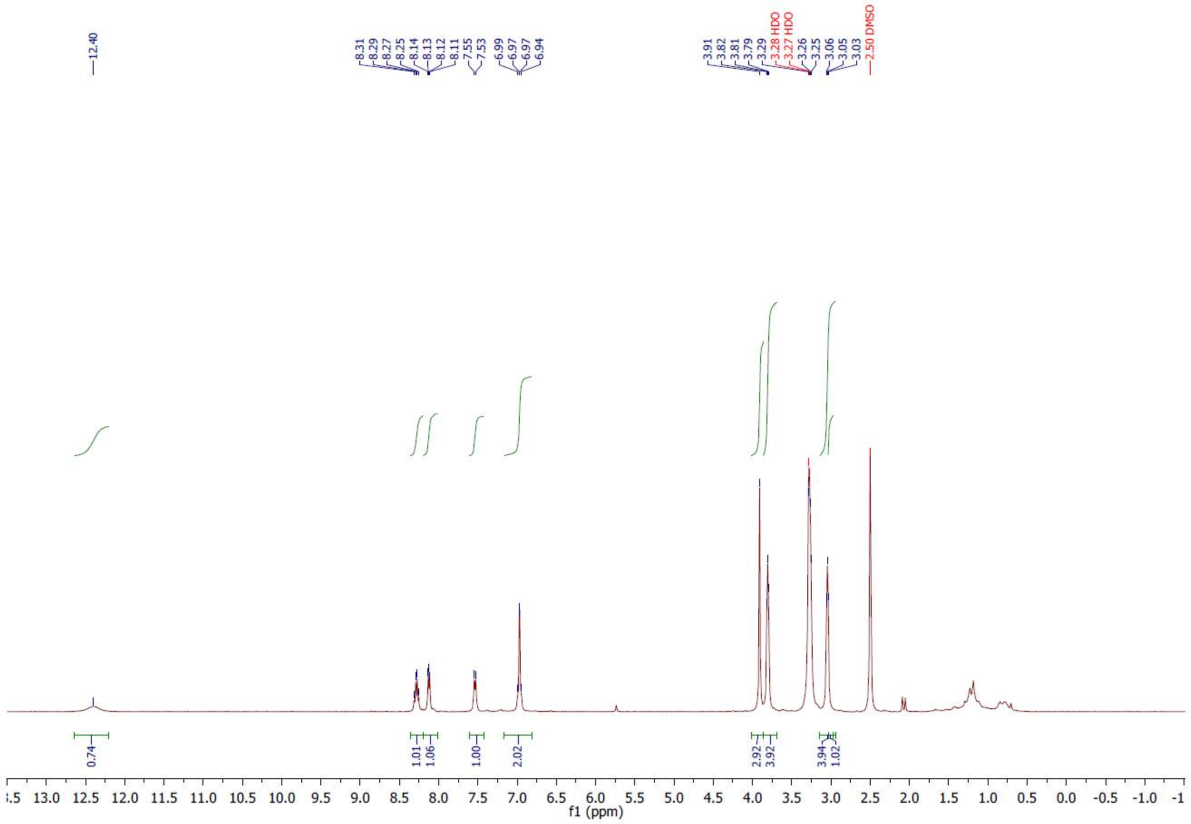

Figure S8: ^{1}H-NMR of **TOZ4**.

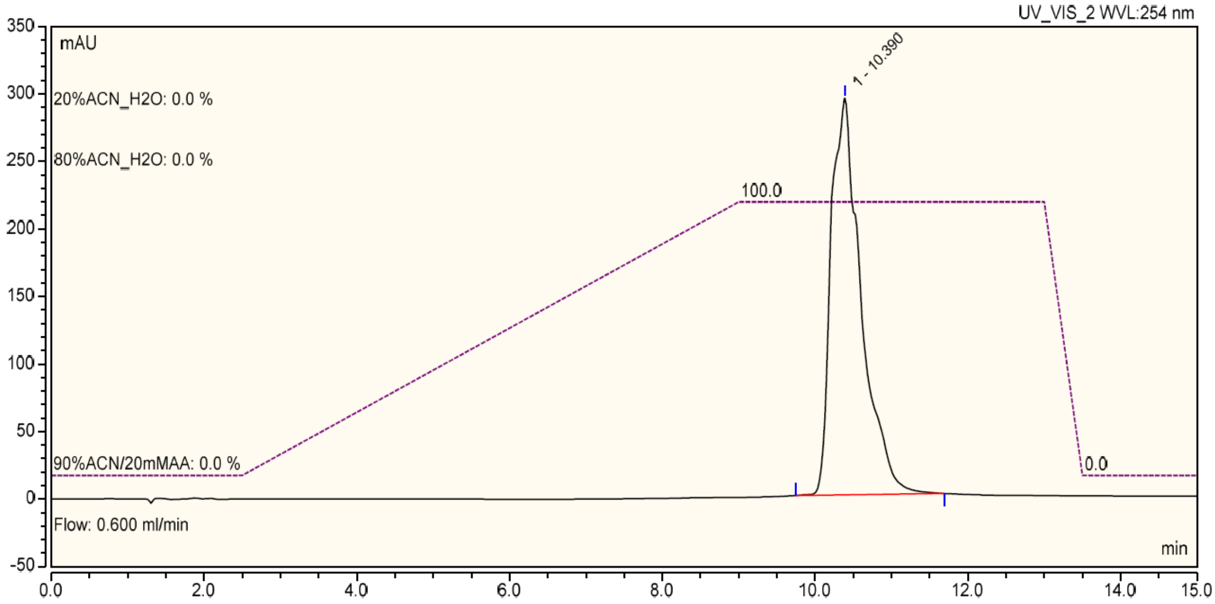

Figure S9: LC-MS chromatogram of **TOZ4**.

4-Fluoro-*N*-(4-methoxy-7-morpholinobenzo[*d*]thiazol-2-yl)benzamide (TOZ5)

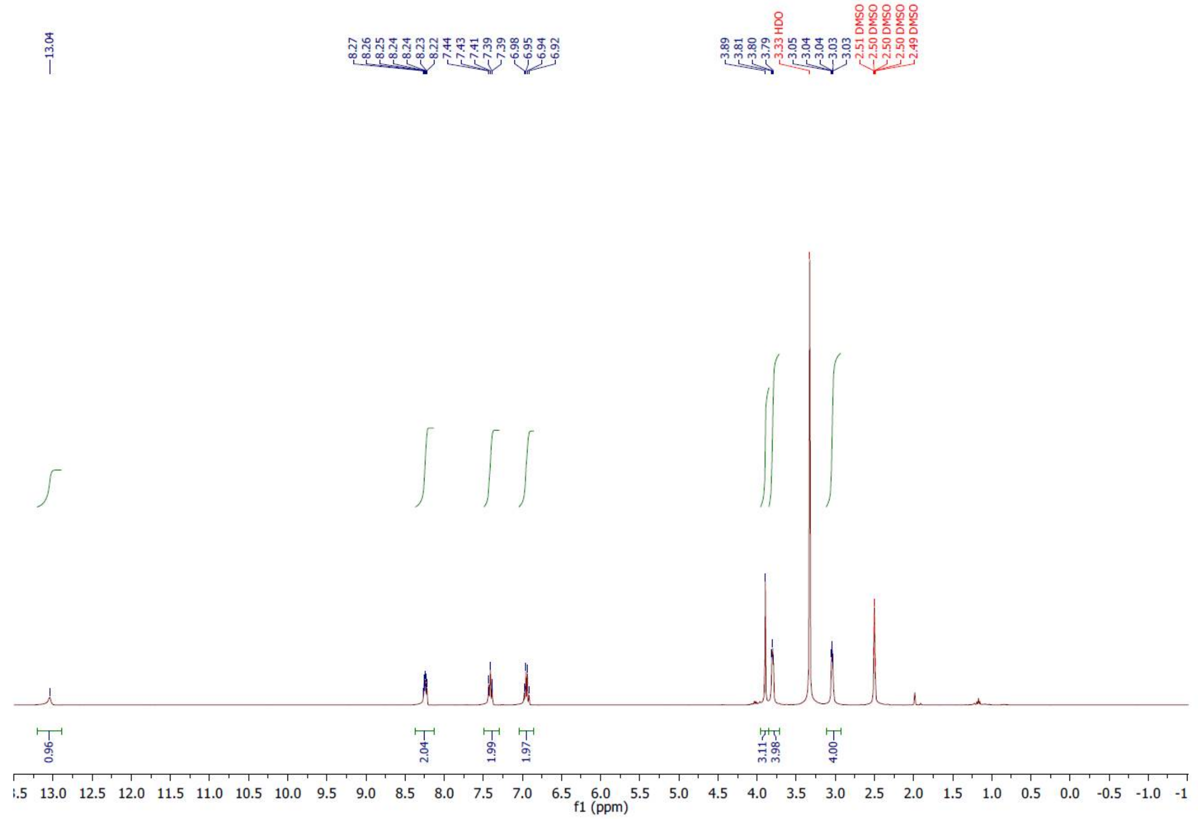

Figure S10: ^{1}H-NMR of **TOZ5**.

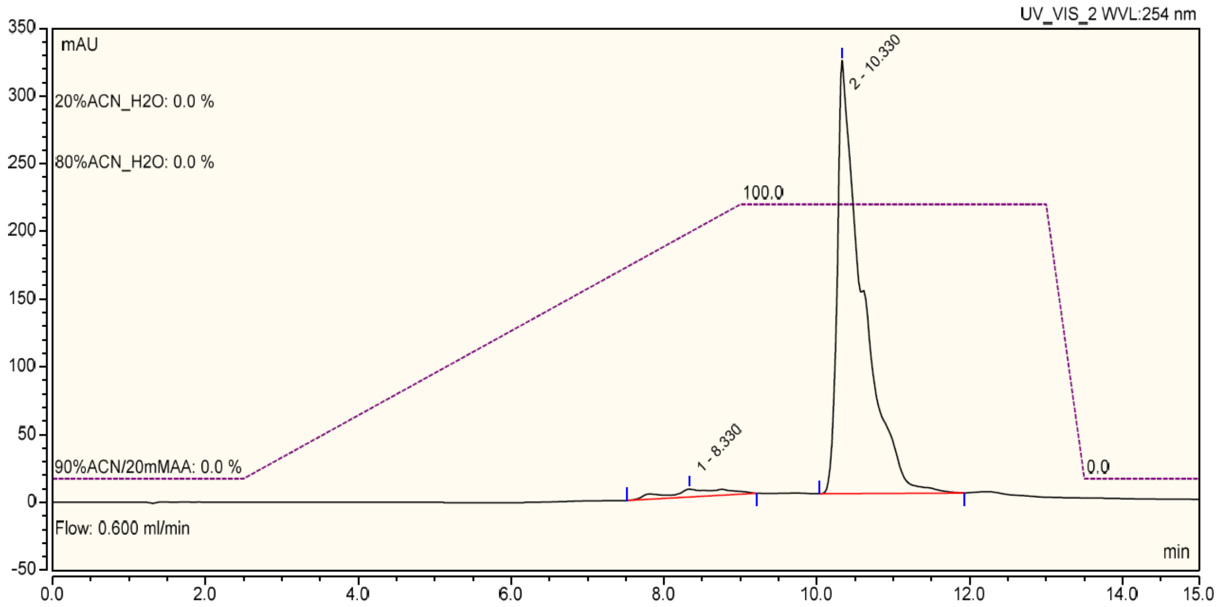

Figure S11: LC-MS chromatogram of **TOZ5**.

2-Fluoro-*N*-(4-methoxy-7-morpholinobenzo[*d*]thiazol-2-yl)benzamide (TOZ6)

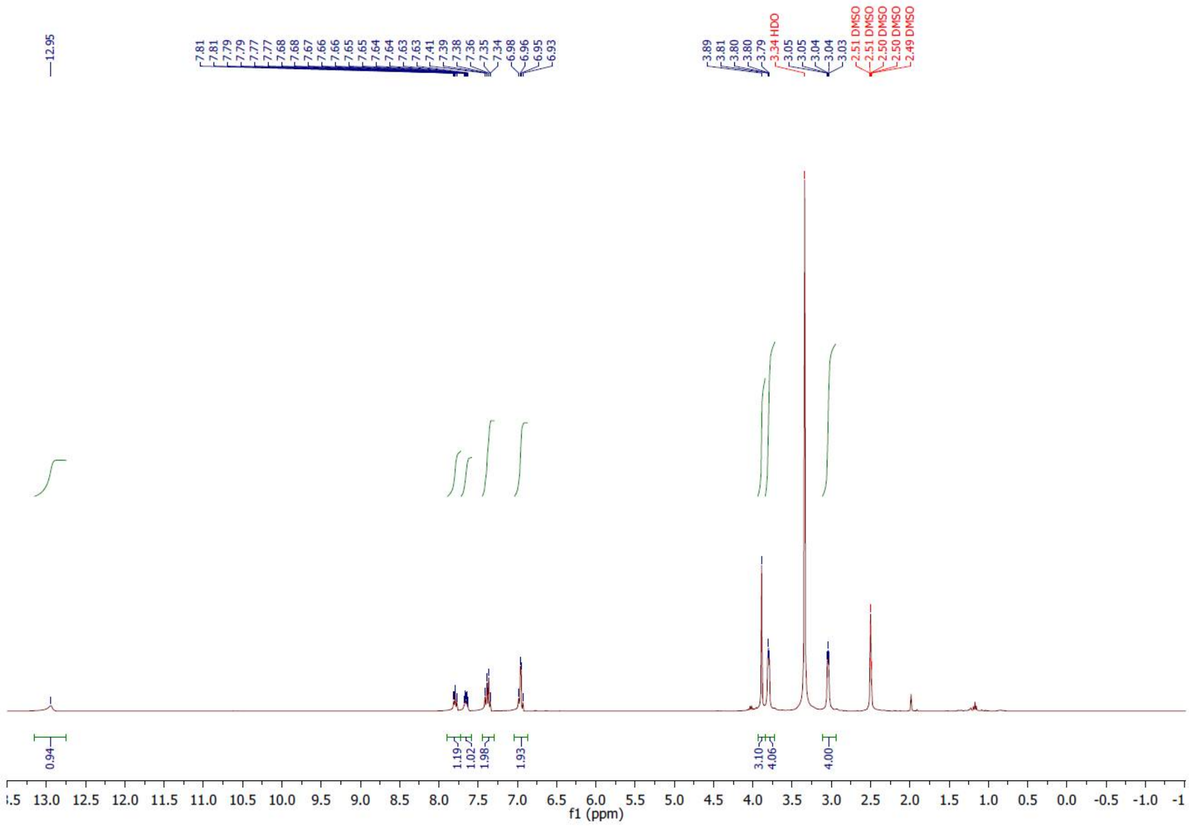

Figure S12: ^{1}H-NMR of **TOZ6**.

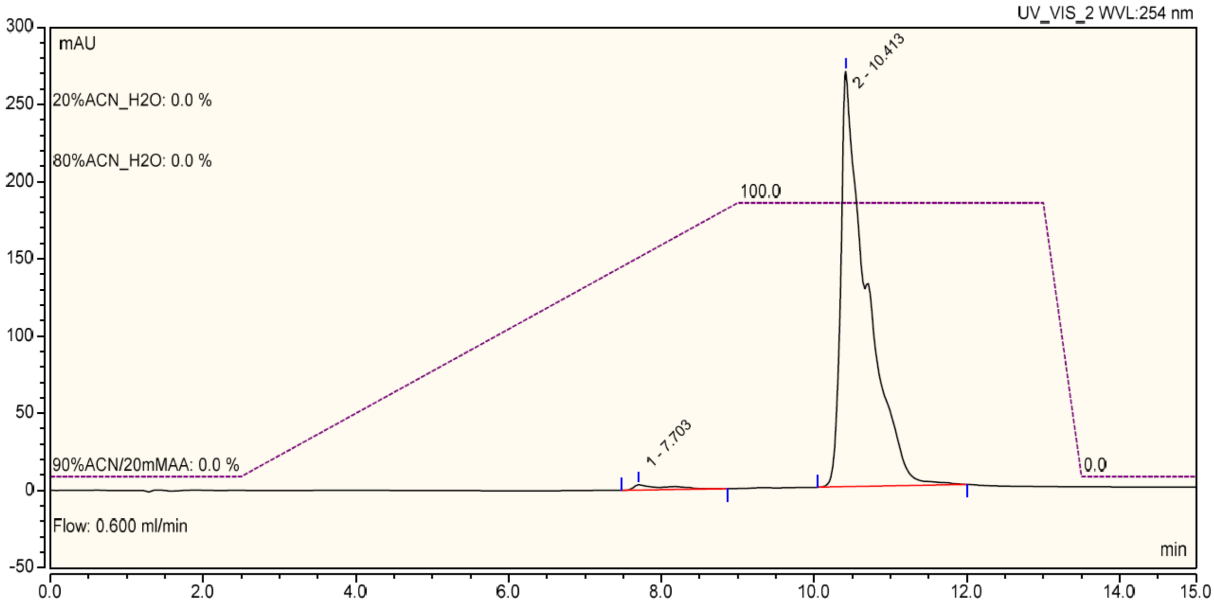

Figure S13: LC-MS chromatogram of **TOZ6**.

4-Fluoro-*N*-(4-methoxy-7-morpholinobenzo[*d*]thiazol-2-yl)-3-nitrobenzamide (TOZ7)

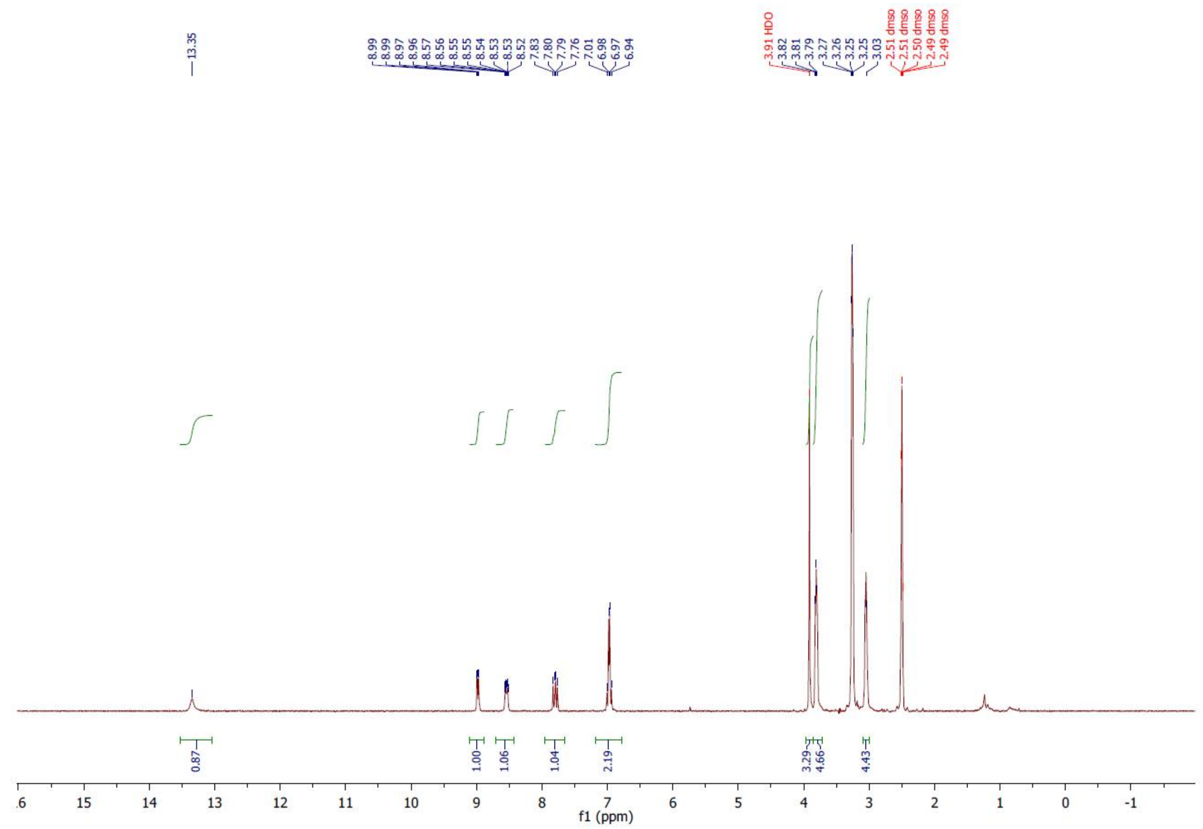

Figure S14: ^{1}H-NMR of **TOZ7**.

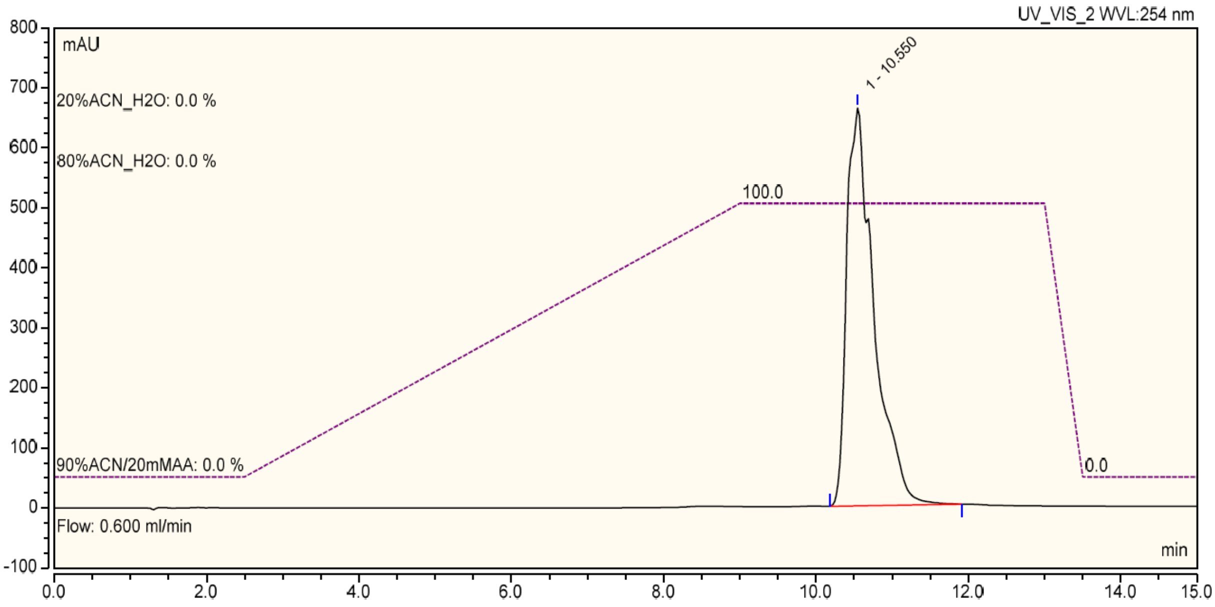

Figure S15: LC-MS chromatogram of **TOZ7**.
